# CHHM: a Manually Curated Catalogue of Human Histone Modifications Revealing Hotspot Regions and Unique Distribution Patterns

**DOI:** 10.7150/ijbs.95954

**Published:** 2024-07-02

**Authors:** Wendong Ma, Xiaofan Ding, Jiajia Xu, Terence Chuen Wai Poon

**Affiliations:** Institute of Translational Medicine, Centre for Precision Medicine Research and Training, MoE Frontiers Science Center for Precision Oncology, Faculty of Health Sciences, University of Macau, Macau, 999078, China

**Keywords:** curated catalogue of human histone modifications (CHHM), human histone modification, histone-DNA crosslink, hotspot region, modification inference

## Abstract

Histone modification is one of the key elements in epigenetic control and plays important roles in regulation of biological processes and disease development. Currently, records of human histone modifications with various levels of confidence in evidence are scattered in various knowledgebases and databases. In the present study, a curated catalogue of human histone modifications, CHHM, was obtained by manual retrieval, evidence assessment, and integration of modification records from 10 knowledgebases/databases and 3 complementary articles. CHHM contains 6612 nonredundant modification entries covering 31 types of modifications (including 9 types of emerging modifications) and 2 types of histone-DNA crosslinks, that were identified in 11 H1 variants, 21 H2A variants, 21 H2B variants, 9 H3 variants, and 2 H4 variants. For ease of visualization and accessibility, modification entries are presented with aligned protein sequences in an Excel file. Confidence level in evidence is provided for each entry. Acylation modifications contribute to the highest number of modification entries in CHHM. This supports that cellular metabolic status plays a very important role in epigenetic control. CHHM reveals modification hotspot regions and uneven distribution of the modification entries across the histone families. Such uneven distribution may suggest that a particular histone family is more susceptible to certain types of modifications. CHHM not only serves as an important and user-friendly resource for biomedical and clinical researches involving histone modifications and transcriptional regulation, but also provides new insights for basic researches in the mechanism of human histone modifications and epigenetic control.

## Background

In eukaryotic cells, DNA is wrapped around histone proteins, forming the fundamental structural unit of chromatins, i.e., nucleosome. The structure of nucleosome usually consists of a segment of DNA and a histone octamer, which is composed of two copies of individual histone proteins H2A, H2B, H3 and H4. The histone protein H1 is located outside the nucleosome. While binding to the nucleosome, H1 binds to the linker DNA between nucleosomes. Instead of being directly exposed to various regulatory proteins, both the transcriptional and replication activities for nucleosomal DNA segments are inherently hindered by the histone proteins. On the other hand, recent studies reveal that different nucleosomal regions are associated with different transcriptional activities, commonly characterized by different sets of modifications on the histone proteins. For example, acetylation of histone H3 on lysine 27 (H3K27ac) is an indicator for active transcription 1. Mono-methylation of histone H3 on lysine 4 (H3K4me1) is associated with active enhancers 2, whereas tri-methylation of histone H3 on lysine 4 (H3K4me3) is associated with promoter-proximal pause-release 3. Multiple modifications work together to form a histone code for regulation of gene transcription.

Histone modifications influence nucleosome unwrapping and stability in turn to regulate transcription, DNA replication and DNA repair 4. Modifications at histone tail regions affect nucleosome unwrapping and stability. Modifications within the nucleosome DNA entry/exit regions affect unwrapping dynamics. Modifications near the nucleosome dyad symmetry axis and at histone-histone interface affect the nucleosome stability. Like epigenetic modifications, histone modifications can be propagated during cell division 5, playing important roles in the development of various types of cells and tissues. Disturbance of this process will inevitably interrupt normal cellular activity and cause abnormal cell phenotypes. Aberrations in the patterns of histone modifications are common in cancers and other degenerative diseases in human.

Elucidation of human histone modifications provides an important foundation for basic, biomedical, and clinical research. With the technical advances, particularly mass spectrometry-based proteomic approaches, more histone modifications at a unique site have been reported. In 2010, a human histone modification database (HHMD) was reported by Zhang Y, et al 6. However, this database has not been updated and is no longer accessible. Although there are a considerable number of currently accessible knowledgebases/databases providing information of identified and inferred histone modifications in human, each of them only provides limited records of histone modifications at different levels of confidence in evidence. Furthermore, there is no database providing a comprehensive comparison of types and sites of modifications among histone variants in a visibly accessible representation based on sequence alignment. To obtain a clear picture of histone modifications, one needs to manually search for the relevant modification information from multiple databases and integrate the retrieved information for summarization and interpretation. In the present study, we manually curated records of histone modifications from 10 public knowledgebases/databases and 3 complementary articles. Modification entries are presented with aligned protein sequences in an Excel file for ease of visualization and accessibility. Moreover, we examined distribution patterns of the curated modification records.

## Materials and Methods

### Sources of human histone modification records

Through literature search, 16 publicly accessible knowledgebases/databases containing records of human histone modifications were identified. After manual comparison of sources of the modification records among them, six knowledgebases/databases (Phosida, IntAct, PRO, HPRD, neXtProt, Signor) were excluded to avoid redundancy. Finally, 10 publicly accessible knowledgebases/databases were included in the present work. Each knowledgebase/database was annotated with a unique capital letter in our curated modification catalogue. They are iPTMnet (annotated with “N”, an integrated resource for protein post-translational modification obtained by using an integrative bioinformatics approach, https://research.bioinformatics.udel.edu/iptmnet/) 7, UniProtKB (annotated with “U”, a knowledgebase for protein sequences annotated with functional information and modifications, https://www.uniprot.org/) 8, PhosphoSitePlus (annotated with “P”, a knowledgebase for modifications, including phospho, acetyl, ubiquityl and methyl groups, https://www.phosphosite.org/) 9, phospho.ELM (annotated with “E”, a database for phosphorylation, http://phospho.elm.eu.org) 10, HISTome2 (annotated with “H”, a knowledgebase for histone modifications, http://www.actrec.gov.in/histome2) 11, GlyGen (annotated with “G”, a computational and informatics resources for glycoscience, https://glygen.org/) 12, GlyConnect (annotated with “L”, a database for glycosylation with primary information, https://glyconnect.expasy.org/) 13, MetOSite (annotated with “M”, a database for methionine oxidation, https://metosite.uma.es) 14, SwissPalm (annotated with “S”, a database for S-palmitoylation, https://swisspalm.org/) 15, and dbSNO (annotated with “D”, a database for cysteine* S*-NitrOsylation, http://dbSNO.mbc.nctu.edu.tw) 16.

Besides, 3 articles 17,18,19 providing complementary records of human histone modifications were also included. Stützer et al. reported UV-induced histone-DNA crosslink sites on human histone H1.4 (annotated with “1”) 17. O'Neil et al. reported a site list for carbamoylation on H3 and H4 induced by phorbol myristate acetate in neutrophil extracellular traps from healthy donors (annotated with “2”) 18. Andrés et al. reported a systematic summarization of modifications on histone H1 from various organisms including human (annotated with “3”) 19. Notably, UV-induced histone-DNA crosslink, carbamoylation, and some records summarized in Andrés et al.'s article have not been collected in the 10 publicly accessible knowledgebase/databases.

### Modification annotation

In general, retrieved modification records are annotated with an abbreviation reflecting the modifying group (e.g., ph for phosphorylation, ac for acetylation). For *N*-terminal acetylation, it is annotated with N-ac regardless of the modified amino acid residue. For acetylation at lysine, it is annotated with ac. For *N*-terminal trimethylation at lysine, it is annotated with N-me3. For monomethylation, dimethylation, trimethylation, and mono/di/trimethylation (i.e., methylation on this site is identified or inferred, but the type of methylation has not been clarified in the source knowledge/database) at non-*N*-terminal lysine, they are annotated with me (K), me2, me3 and meX (K), respectively. For monomethylation, symmetric dimethylation, asymmetric dimethylation, sym/asym dimethylation (i.e., dimethylation on this site is identified or inferred, but the type of methylation has not been clarified in the source knowledgebase/database), and mono/dimethylation (i.e., methylation on this site is identified or inferred, but the type of methylation has not been clarified in the source knowledge/database) at arginine, they are annotated with Nω-me, sym me2, asym me2, sym/asym me2, and meX (R), respectively. For monomethylation at glutamine, it is annotated with me (Q). For histone-DNA crosslink at base T and at base C, they are annotated with crosslink_T and crosslink_C, respectively.

### Amino acid sequence alignment and inference of modification on histone variants

For each histone family (e.g., H1), protein sequences of the variants were retrieved from UniProt, and the sequences were first aligned using Clustal Omega (1.2.4) (default settings, https://www.ebi.ac.uk/Tools/msa/clustalo/) 20 and subsequently adjusted by manual inspection. After amino acid sequence alignment of the variants, the modification records at each aligned amino acid residue are compared. For a specific type of modification (e.g., phosphorylation), if there are more than one variant having a modification record at an aligned amino acid residue (e.g., serine), an inferred modification of the same type was assigned to other variants at the same aligned amino acid residue containing a side chain with the same/compatible functional group (e.g., serine or threonine) that allows the occurrence of the same modification.

### Assessment of confidence level in evidence

All retrieved modification records were manually inspected and classified into 3 categories based on the confidence level according to experimental evidence. The highest level of confidence CL3 was given to those modifications identified/confirmed by low-throughput experimental methods, i.e., typical experiments generating solid evidence. A middle level of confidence CL2 was given to those only identified by high-throughput experimental methods, e.g., mass spectrometry-based bottom-up proteomic experiments. The lowest level of confidence CL1, including CL1a and CL1b, was given to those modifications identified by inference based on protein sequence similarity.

These confidence levels of the modification records were defined as follows:

i. For records with CL3 (highest level), they were generated by low-throughput experimental identification (LTP).

ii. For records with CL2, they were only generated by high-throughput experimental identification (HTP).

iii. For records with CL1a, they were identified by inference based on protein sequence similarity and recorded in at least one of the 10 knowledgebases/databases (ISD).

iv. For records with CL1b, they were identified by inference based on protein sequence similarity and only identified in the present study (ISS).

For modification sites, we also classified them into 3 categories based on the confidence level according to experimental evidence. As different types of modification could be identified at the same site of a histone protein, the confidence level of a modification site was assigned to be equal to the highest confidence level among the modification records associated with the same amino acid residues.

## Results and Discussion

### Summary of modification entries in CHHM

Eleven variants of human H1, 21 variants of human H2A, 21 variants of human H2B, 9 variants of human H3, and 2 variants of human H4 were identified to have modifications recorded. The identified human histone variants are summarized in Table S1. In total, 4364 nonredundant modification records were retrieved from the 10 modification knowledgebases/databases and the 3 complementary articles. These records cover 31 types of histone modifications and 2 types of UV-induced histone-DNA crosslinks. These records cover 2 subtypes for acetylation and 8 subtypes for methylation. Moreover, records of 9 novel modifications emerging in the recent years are also identified, including crotonylation 21, dopaminylation 22, glutarylation 23, *β*-hydroxybutyrylation 24, 2-hydroxyisobutyrylation 25, lactylation 26, malonylation 27, succinylation 28, and UFMylation 29.

Among these 4364 curated records, 2137 records are supported by findings generated in low-throughput experiments and were assigned to have a confidence level of CL3, i.e., the highest confidence level; 1430 records are supported by findings only discovered in mass spectrometry-based high-throughput experiments and were assigned to have a confidence level of CL2; 797 records are inferred based on protein sequence similarity and were assigned to have a confidence level of CL1a. Through protein sequence alignment and comparison of sidechain functional groups of the aligned amino acid residues, we further identified 2248 modification candidates through inference, and they were assigned to have a confidence level of CL1b. Together with the modification candidates inferred by us, there are a total of 6612 nonredundant modification entries in the final curated catalogue of human histone modifications (CHHM). Most of CL3 modifications are identified in H2B. After normalizing the modification entry counts with the total number of the amino acid residues from all protein variants within a histone protein family, the highest amount of CL3 modifications are associated with H4, followed by H2B. The least amount of CL3 modifications are associated with H1, indicating more wet laboratory work for modification discovery is needed for H1. The distribution of the modification entries of different confidence levels among the histone protein families is summarized in Table 2.

### Visualization of CHHM

**Figure 1:**
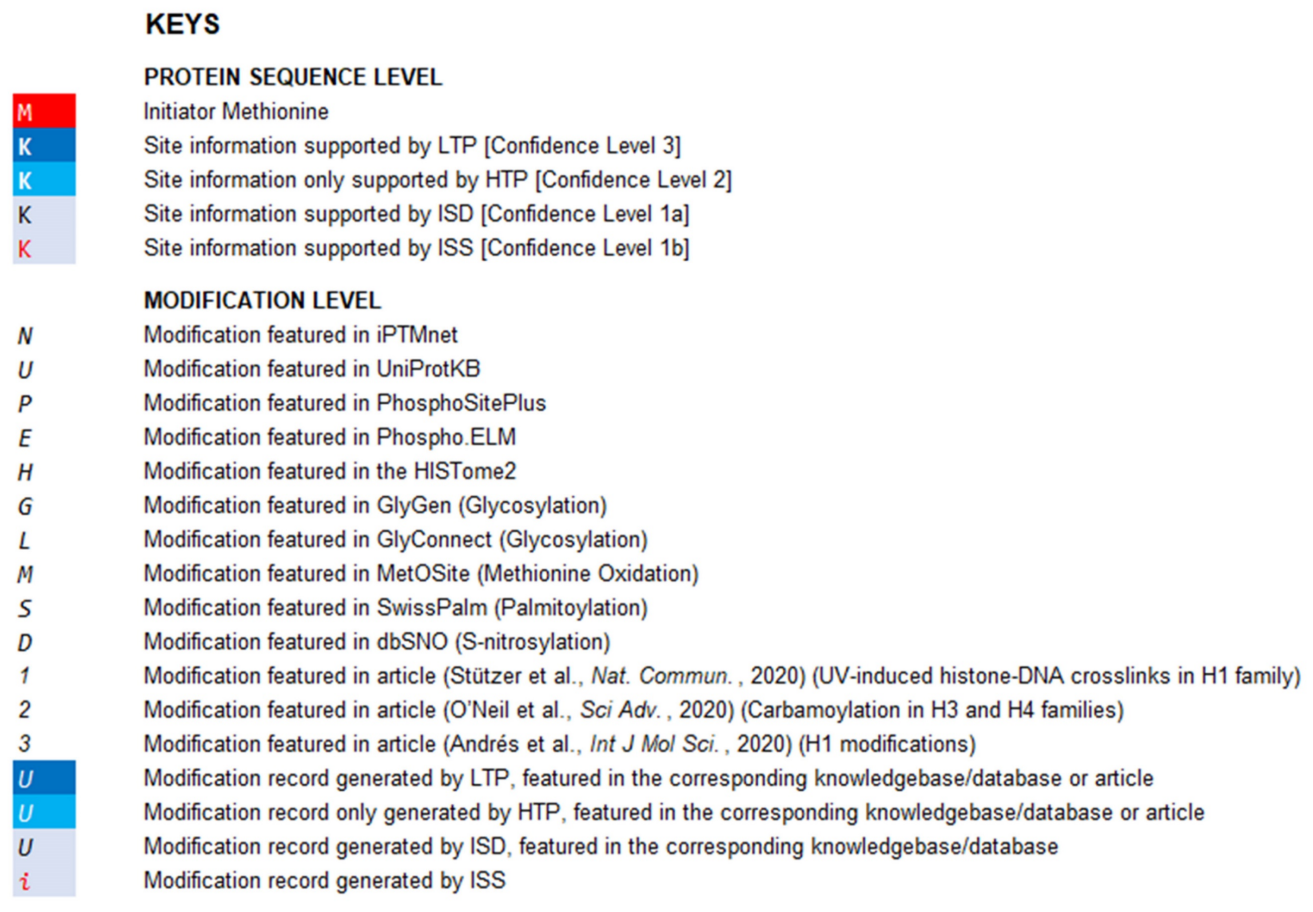
Key table in CHHM. The curated modification entries in CHHM are delivered at two levels: protein sequence level and modification level. At protein sequence level, capital letters denote amino acid symbols (e.g., M for methionine, K for lysine). At modification level, capital letters or numbers in italic denote the source of a modification entry (e.g., *N* for a modification featured in iPTMnet; *2* for a modification featured in O'Neil et al., *Sci adv*., 2020). At both protein sequence level and modification level, font color and background color denote the confidence level as shown in the key table. Besides, the initial methionine is indicated with a red background color.

**Figure 2:**
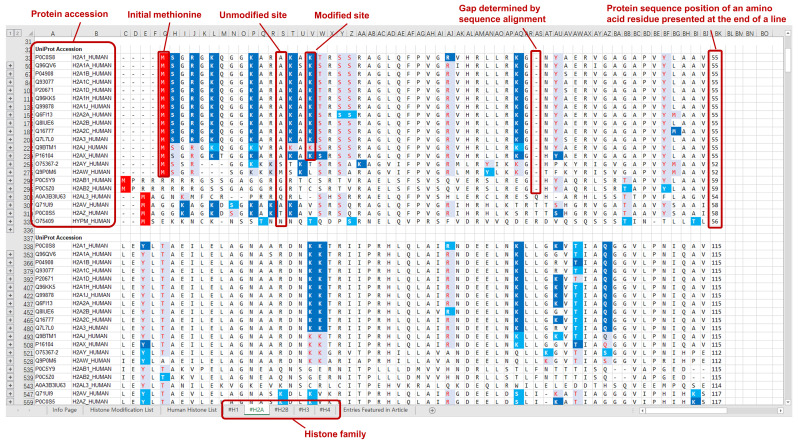
An example page of CHHM providing information of the modification entries at protein sequence level. At protein sequence level, aligned amino acid sequences of the histone variants within a family are provided. Gaps in protein sequence are determined by sequence alignment. The confidence of the amino acid residue being a modification site is indicated by the confidence level of the modification entry with the highest confidence of evidence among all entries associated with the amino acid residue. The degree of confidence is denoted by a combination of the background color and the color of the amino acid symbol (Figure 1 for details). The initial methionine is highlighted with a red background color.

**Figure 3:**
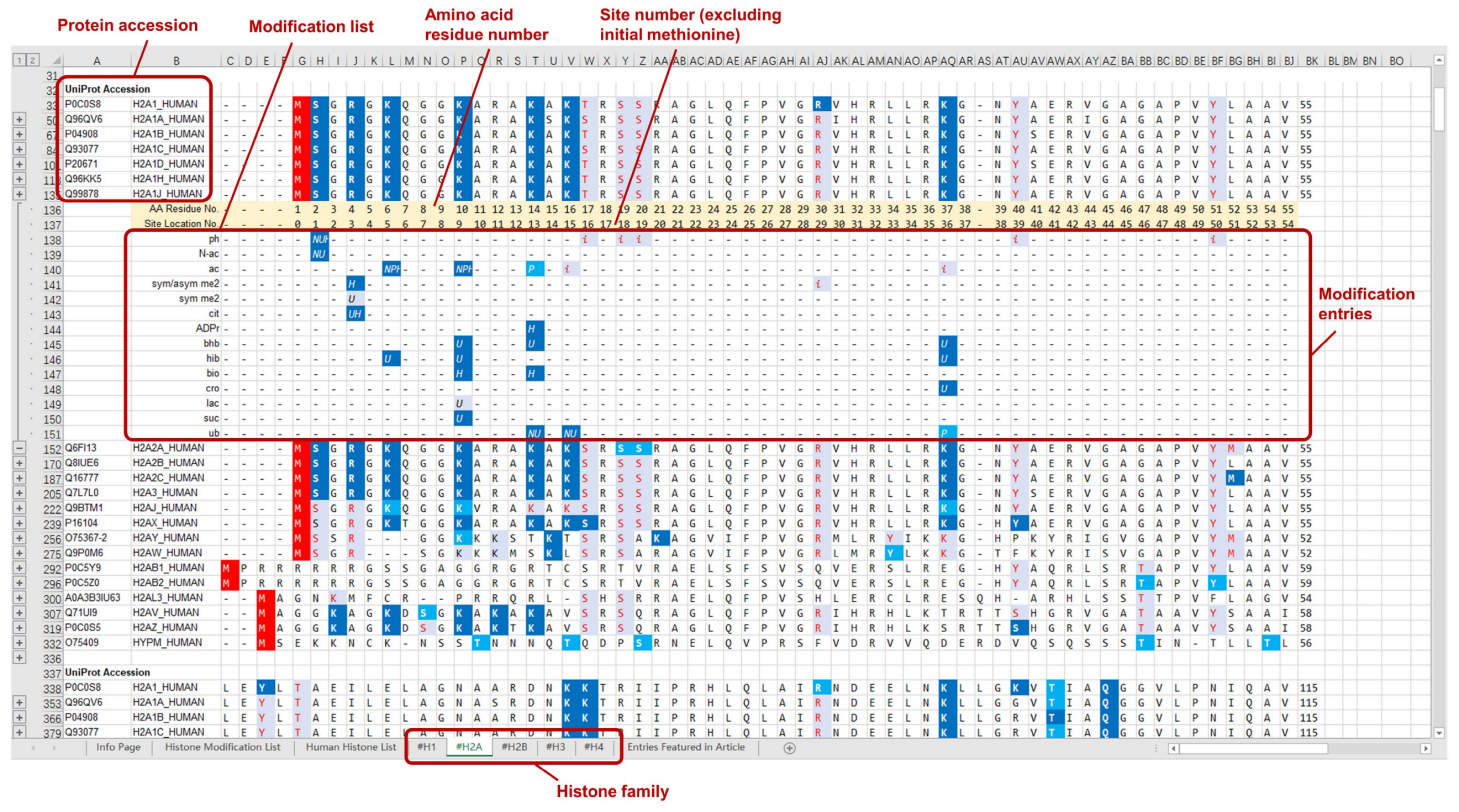
An example page of CHHM providing information of the modification entries at modification level. To visualize the modification entries for each amino acid residue, clicking the “+” button on the left of the consecutive line below the amino acid sequence of a histone variant will give the information of individual modification entries, including type, localization, source, and confidence level. For each modification entry, its type information is indicated at the beginning of each line of entries with the corresponding abbreviation. The italic letters/numbers indicate the sources of the entries, i.e., knowledgebase/database or article. The combination of font color and background color denotes the highest confidence level of the retrieved records for each entry (Figure 1 for details).

The curated modification entries in CHHM are delivered at two levels: protein sequence level and modification level. For the ease of accessibility and usage, the modification entries of individual histone families (H1, H2A, H2B, H3, and H4) are presented on 5 separate worksheets in an Excel file and provided as a supplementary material of this article. Each worksheet includes the aligned sequences of the histone variants. Modification entries of each histone member are indicated underneath the aligned amino acid sequence. The confidence level of each modification is indicated with different colors. Example pages of CHHM are given in Figures 1, 2, and 3.

### Prevalence of amino acids with modification

Most (78.3%) of the experimentally found modifications (CL2 and CL3) occur at lysine residues regardless of the histone families (H1: 74.1%, H2A: 78.2%, H2B: 81.4%, H3: 72.4%, H4: 83.3%). Serine, threonine, arginine, and tyrosine have relatively higher susceptibility to modifications. Other minor modification sites included protein *N*-termini, methionine, cysteine, glutamine, glutamic acid, asparagine, and proline. The prevalences of various amino acids with modification in different histone families are summarized in Figure S1.

### Distribution pattern of modification hotspots

**Figure 4:**
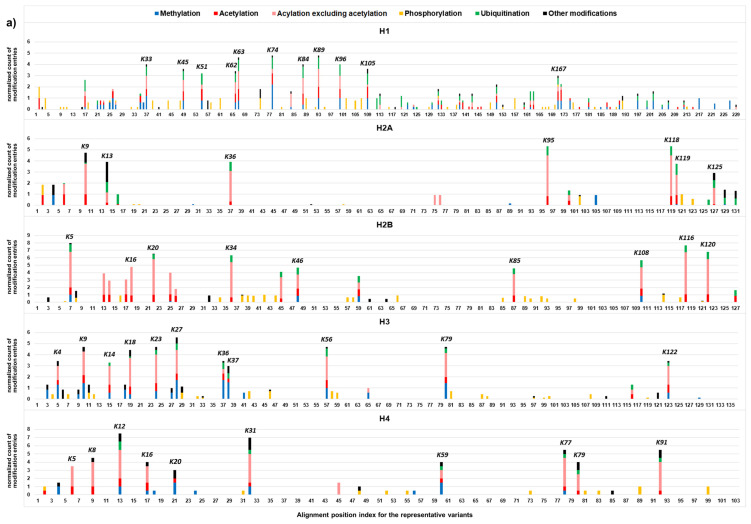

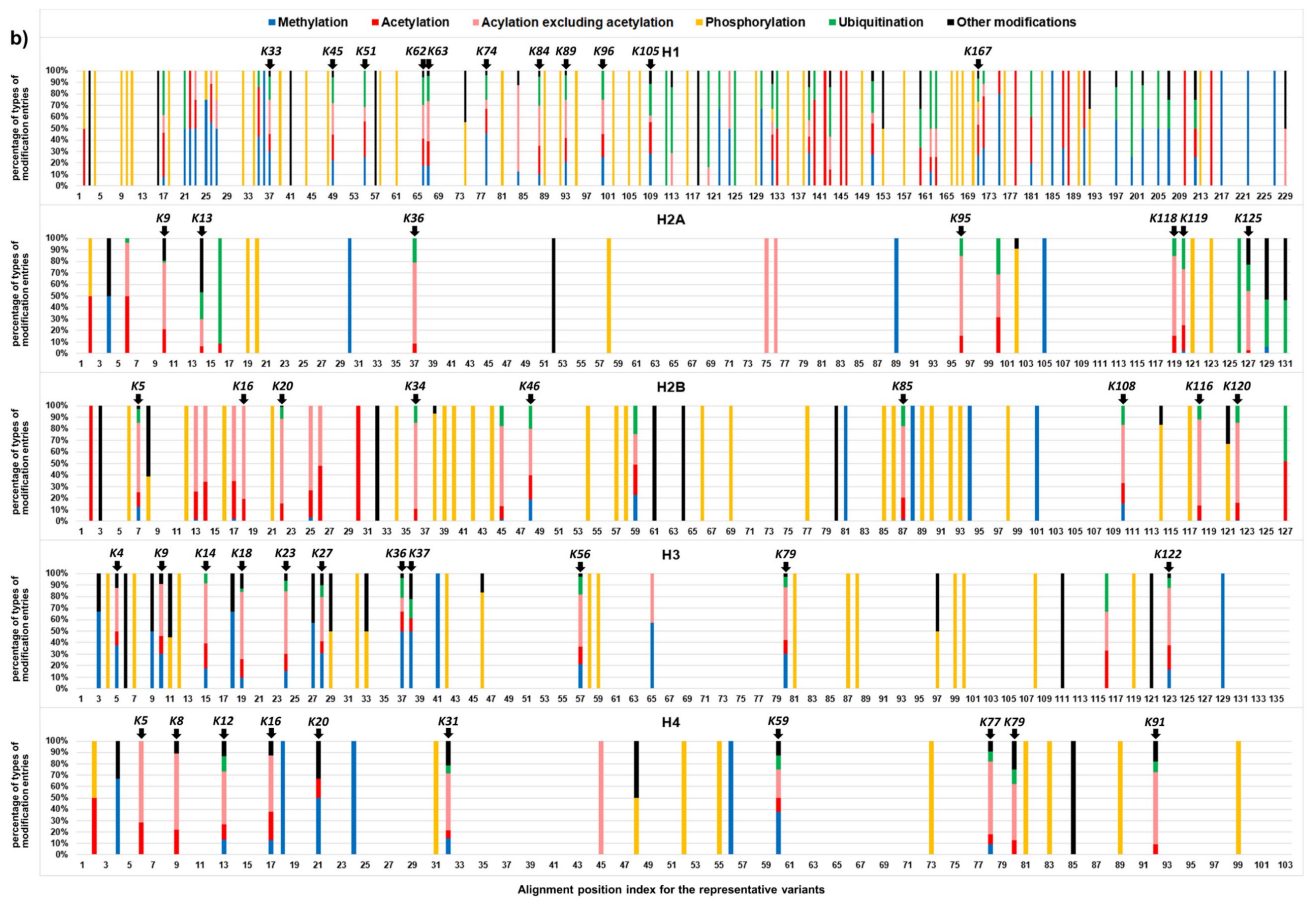
Localization of human histone modifications in representative variants of histone family H1, H2A, H2B, H3, and H4 at confidence levels of CL2 to CL3. Histone variants with high sequence homology were selected as the representative variants (Table S2). Five major types of modifications, methylation, acetylation, acetylation excluding acylation, phosphorylation, and ubiquitination were indicated in different colors. The other types of modifications are indicated in black. The *x*-axis refers to the alignment position index for the representative variants in a histone family. **a**) The *y*-axis refers to the normalized count of modification entries retrieved for all representative variants at an alignment position. A normalized count of modifications was calculated by dividing the count of modification entries by the count of aligned amino acids from all representative variants at an alignment position (gaps were not counted). Hotspot sites are highlighted with labels describing the corresponding modified site on a selected variant sequence for reference. For H1, H2A, H2B, H3, and H4 families, the selected variants are, P10412|H14_HUMAN, P0C0S8|H2A1_HUMAN, P33778|H2B1B_HUMAN, P68431|H31_HUMAN, and P62805|H4_HUMAN, respectively. **b**) The *y*-axis refers to the percentage of different types of modification entries relative to the total number of modification entries at an alignment position.

Localization of curated histone modifications (CL2 to CL3) in the representative variants of histone family H1, H2A, H2B, H3, and H4 (i.e., variants with high sequence homology, Table S2) are summarized in Figure 4. Most of the modifications are localized at specific amino acid residues of the histone proteins, indicating the presence of the modification hotspots and hotspot regions. For H1 protein, lysine hotspots are mainly located to K16-K109 and K158-K168. In core histone families (H2A, H2B, H3, and H4), lysine hotspots are clustered in *N*-terminal histone tail and *C*-terminal histone tails with relatively more at the *N*-terminal tail. For phosphorylation, the modification sites appear to be more evenly distributed within the histone proteins, instead of clustered at the tail regions. This may suggest that phosphorylation plays a unique role in epigenetic control.

The lysine hotspots are consistent with the locations of the histone modifications that play vital roles in various biological processes. For H1, the highest numbers of modification entries were identified at K63, K74 and K89. RNF8/RNF168-mediated ubiquitination at K63 regulates DNA double-strand break repair 30. K9, K13, K36, K95, K118, K119 and K125 are the major modification hotpots on H2A. Ubiquitination by RNF168 at H2AK13, ubiquitination by PRC1 at H2AK118/K119, and ubiquitination by BRCA1/BARD1 at H2AK125 are associated with the response to DNA damage 31. K5, K20, K34, K108, K116 and K120 are the top 6 modification hotpots on H2B. The highest number of modification entries was identified at K5. Methylation at H2BK5 is linked to gene activation 32. MAP3K4/CBP-mediated acetylation at H2BK5 controls epithelial-mesenchymal transition in trophoblast stem cells 33. Higher levels of H2BK120 acetylation were observed in hepatocellular carcinoma associated with poor survival 34. RNF20/RNF40-mediated ubiquitination at H2BK120 is also associated with the DNA damage response 31. K9, K18, K23, K27, K56 and K79 are the top 6 modification hotspots on H3. The highest number of modification entries was identified at K27. H3K27 acetylation is a well-known mark for active enhancers 35, whereas H3K27 trimethylation is a repressive histone mark 32, 36. They antagonize each other and play important roles in development and cell differentiation 37. H3K56 acetylation interacts with Oct4 to promote pluripotency of embryonic stem cells 38. Dot1/DOT1L-mediated H3K79 methylation are involved in transcriptional regulation, cell cycle regulation, and the DNA damage response 39. K12, K32, K77 and K91 are the top 4 modification hotspots on H4. The highest number of modification entries was identified at K12. Acetylation regulation at H4K12 plays important roles in fertilization and embryonic development 40, 41. Glutarylation at H4K91 regulates chromatic dynamics 42.

### Uneven distribution of various types of modification across histone families

**Figure 5:**
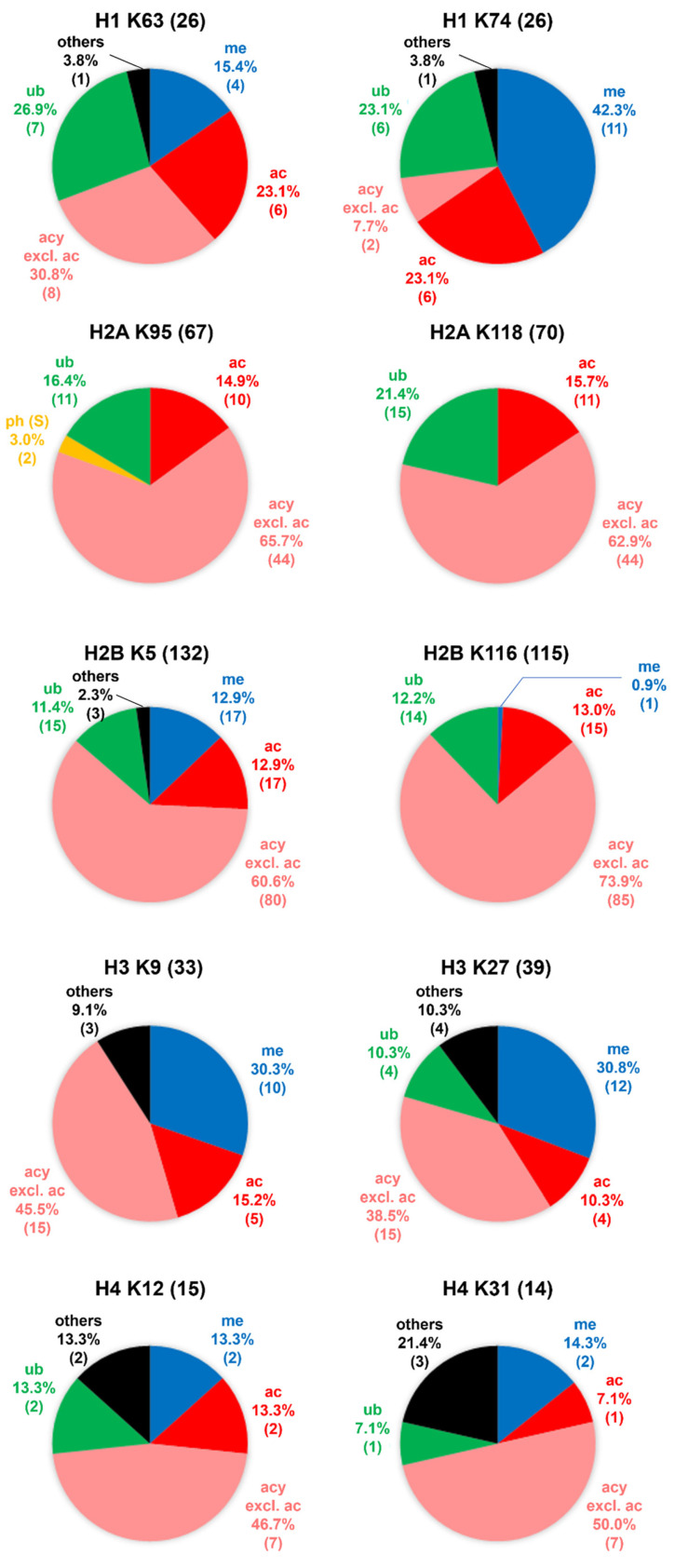
The distribution patterns of various types of modification entries at representative sites among all variants in human histone families H1, H2A, H2B, H3, and H4, at confidence levels of CL2 to CL3. For each histone family, the top 2 hotspots (i.e., two sites with highest numbers of modification entries) are chosen as the representative sites. The abbreviations of modification types are consistent with those in Table 1. Except acetylation, all modifications belonging to the acylation family are grouped together and annotated as “acy excl. ac”. The modification entry counts are given in brackets. In the pie chart for H2A K95, ph (S) refers to phosphorylation at serine, which was identified on Q71UI9|H2AV_HUMAN and P0C0S5|H2AZ_HUMAN at the aligned site. In these two variants, the amino acid residue is serine instead of lysine at the aligned site.

**Figure 6:**
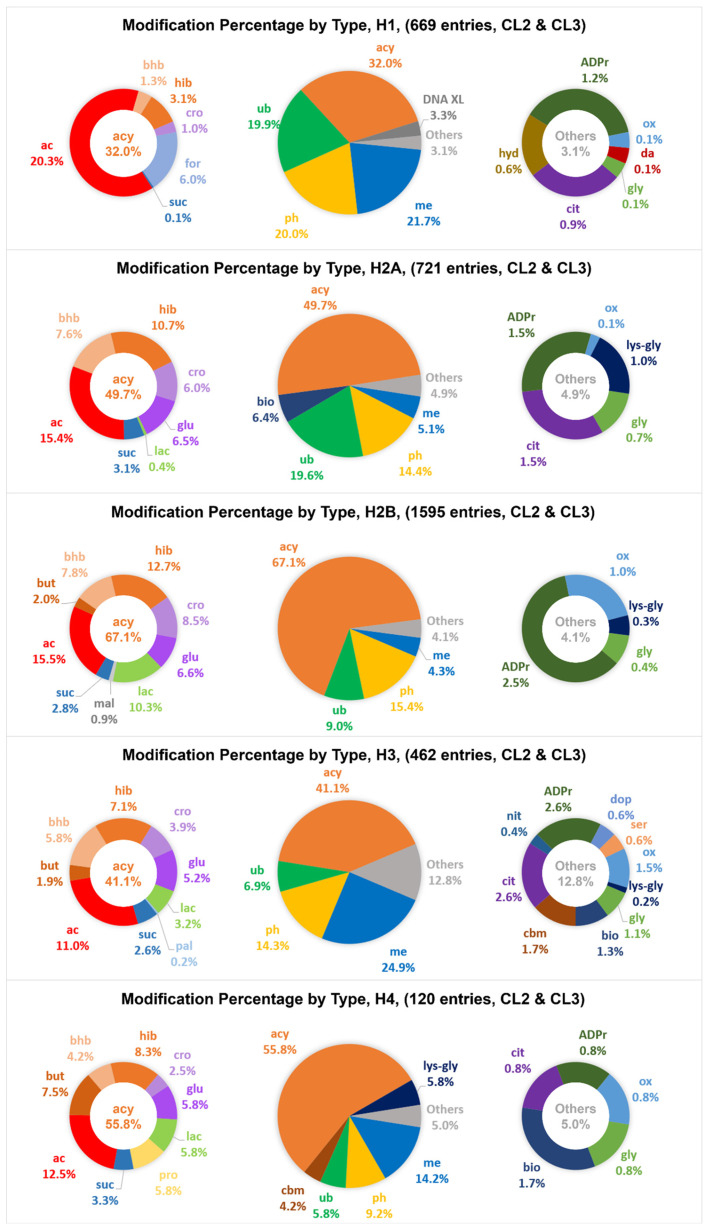
The distribution patterns of various types of modification entries among human histone families H1, H2A, H2B, H3, and H4, at confidence levels of CL2 to CL3. The abbreviations of modification types are consistent with those in Table 1. The detailed distribution of various types of acylation entries is provided on the left, and the detailed distribution of other types of modification entries is provided on the right.

Most amino acid residues of the modification sites are well conserved among the variant members of a histone family. This results in well separated locations for modifications that require a distinct functional group from an amino acid residue for formation (Figure 4), for example, phosphorylation requiring the hydroxyl group of serine/threonine/tyrosine and ubiquitination requiring the *ε*-amino group of lysine. Most of the CL2/CL3 phosphorylation entries were exclusively identified at serine, threonine and tyrosine and scattered over the entire protein sequence, whereas the CL2/CL3 entries of methylation, acetylation, and ubiquitination were predominantly identified at the lysine hotspots. In general, within a histone family, the distribution patterns of the CL2/CL3 modifications at the lysine hotspots (Figure 5) are consistent with the overall distribution patterns of the CL2/CL3 modifications at the protein level (Figure 6). Across the histone families, various types of CL2 and CL3 modifications are unevenly distributed (Figure 6).

In detail, at the protein level of H1, methylation, acetylation, ubiquitination, and phosphorylation are the major types with similar contributions (81.9% of CL2/CL3 modification entries in total). Consistently, acetylation, and ubiquitination are the major modifications with similar contributions at the H1 lysine hotspots. At the protein level of H2A and H2B, acetylation, ubiquitination, and phosphorylation have similar contributions, and contribute to 49.4% and 39.9% of the CL2/CL3 modification entries in total, respectively. Similarly, acetylation and ubiquitination are the predominant modifications at the H2A/H2B lysine hotspots. Noticeably, methylation also serves as a key modification at some H2B lysine hotspots, such as H2BK5. Interestingly, biotinylation is mainly observed in H2A and contributes to 6.4% of the CL2/CL3 modification entries. For H3 and H4, at the protein level of H3 and H4, methylation is the predominant type (24.9% and 14.2% of CL2/CL3 modification entries, respectively), followed by acetylation and phosphorylation; likewise, at the lysine hotspots, methylation is the predominant modification, followed by acetylation. Noteworthily, ubiquitination contributes to considerable amount of the CL2/CL3 modification entries (6.9% and 5.8%, respectively) of H3 and H4. Except the lysine hotspots near the protein N-terminus, ubiquitination has a contribution comparable to acetylation at most H3/H4 lysine hotspots, such as H3K27, H4K12, H4K31, etc. Despite uneven distribution of the modifications across the histone families, acetylation, methylation, phosphorylation, and ubiquitination contribute to a substantial portion of CL2 and CL3 modification entries in all histone families.

It is important to note that acetylation, butyrylation, crotonylation, formylation, glutarylation, *β*-hydroxybutyrylation, 2-hydroxyisobutyrylation, lactylation, malonylation, *S*-palmitoylation, propionylation, and succinylation belong to the acylation modification family. When considering all these 12 acylation species together (including acetylation), acylation modifications are the predominant modifications among all histone families as well as at most of the lysine hotspots. As acylation modifications were shown to be associated with energy metabolism 43, the predominance of the acylation modification entries suggests that cellular metabolic status plays a very important role in epigenetic control.

### Uneven occurrence of representative modifications in nucleosome

**Figure 7:**
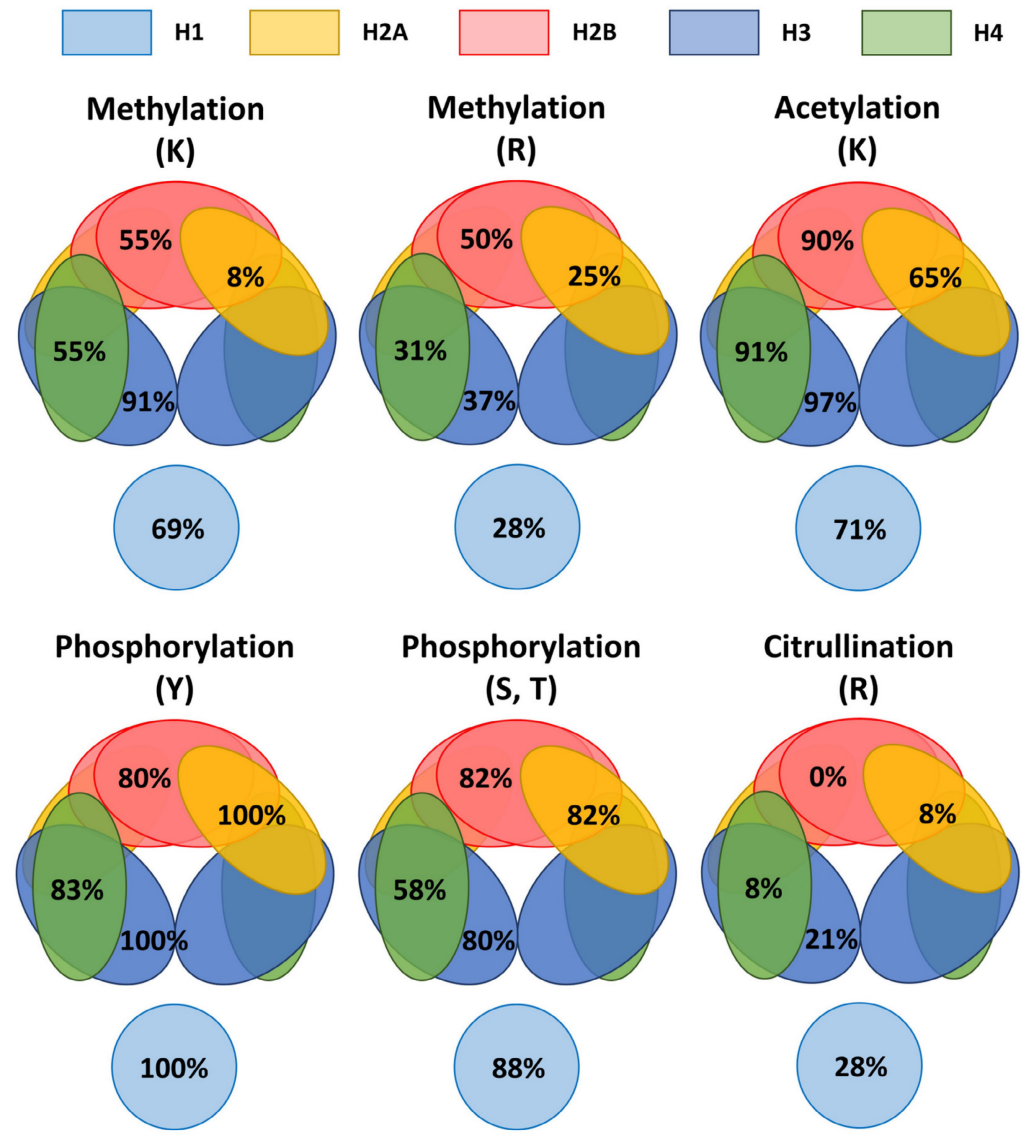
A schematic representation of the occurrence percentages for representative modifications at specific amino acid residues in the nucleosome. Methylation, acetylation, phosphorylation, and citrullination are selected as the representative modifications. Modification entries at confidence levels of CL1 to CL3 were included in the calculation. The occurrence percentage (e.g., methylation at lysine in H3) at a specific amino acid residue of a histone family is the percentage of total count of a given amino acid residue (e.g., lysine) from all variants of a histone family (e.g., H3) having an entry of a given type of modification (e.g., methylation). For example, for methylation at lysine on H3, 91% of occurrence means 91% of lysine residues from all H3 variants having a record of methylation. For phosphorylation at tyrosine on H2A, 100% of occurrence means all tyrosine residues from all H2A variants having a record of phosphorylation.

Methylation at lysine, methylation at arginine, acetylation at lysine, phosphorylation at serine or threonine, phosphorylation at tyrosine, and citrullination at arginine were selected as representative modifications for exploring their occurrence patterns in the nucleosome. We calculated the occurrence percentage for a specific modification (CL1 to CL3) at a specific amino acid residue (e.g., methylation at lysine) of all variants in individual histone families and compared. The results are summarized in Figure 7. For methylation at lysine, it happens at 91% on H3, but only at 8% on H2A. For methylation at arginine, it happens at the highest percentage on H2B (50%) that is two times of the percentage on H2A (25%). For acetylation at lysine, it happens at over 90% on H2B, H3 and H4, but only at 65% on H2A. For phosphorylation at tyrosine, it happens at ≥80% of all histone families. For phosphorylation at serine or threonine, it happens at ≥80% of H1, H2A, H2B and H4, but only at 58% of H4. For citrullination at arginine, it happens at much lower percentage compared to other modifications. It happens at over 20% at H1 and H3 and at 8% on H2A and H4, but it has not been found on H2B. These results reveal uneven distributions of the examined modifications among the histone families. Furthermore, the uneven patterns are different for different types of modifications. H2A appears to be the least susceptible to methylation either at lysine (8%) or arginine (25%). H2B appears to be not susceptible to citrullination at arginine (0%). H3 appears to be the least susceptible to phosphorylation at serine or arginine (58%). These results suggest uneven occurrence of various types of modifications in the nucleosome.

A preferred occurrence of various types of modifications among the histone families may reflect the binding points and working directions of enzymes, structural affinity, spatial hindrance, modification competition, and perhaps, proximity for crosslinking. To answer whether these uneven patterns of the examined modifications among the histone families are caused by biological factors or experimental biases, more investigations are needed. Concerning citrullination on H2B, we are surprised that citrullination records have not been found among the 10 knowledgebases/databases. Citrullinated H2B was reportedly identified 44. More studies should be conducted to clarify whether citrullination could happen on H2B.

## Conclusion

A curated catalogue of human histone modifications, CHHM, was obtained by manual curation of modification records from 10 knowledgebases/databases and 3 complementary articles. CHHM contains 6612 nonredundant modification entries covering 31 types of modifications (including 9 types of emerging modifications) and 2 types of histone-DNA crosslinks, that were identified in 11 H1 variants, 21 H2A variants, 21 H2B variants, 9 H3 variants, and 2 H4 variants. Modification entries are presented with aligned protein sequences in an Excel file for ease of visualization and accessibility. Each worksheet is password-protected. Acylation modifications contribute to the highest number of modification entries in CHHM, supporting an important role of cellular metabolic status in epigenetic control. CHHM reveals modification hotspot regions and unique distribution patterns across the histone families. CHHM not only serves as an important and user-friendly resource of human histone modifications, but also provides new insights for understanding the mechanisms of human histone modifications and epigenetic control.

## Supplementary Material

Table S1. Summary of identified human histone variants. Table S2. The list of representative human histone variants for hotspot analysis. Figure S1. The distribution patterns of various sites of modifications among human histone families H1, H2A, H2B, H3, and H4, at confidence levels of CL2 to CL3, (PDF).

User manual of Curated Catalogue of Human Histone Modifications (CHHM), (PDF).

Curated Catalogue of Human Histone Modifications (CHHM), (XLSX), Password: CHHM.

## Figures and Tables

**Table 1:** Summary of 31 types of modifications and 2 types of crosslinks included in the curated catalogue of human histone modification, CHHM. For acetylation and methylation, annotations of their subtypes are presented. Asterisk (*) denotes the situation in which the methylation on lysine is identified but the type of methylation is not clear. Double asterisk (**) denotes the situation in which the dimethylation on arginine is identified but the type of dimethylation is not clear. Triple asterisk (***) denotes the situation in which the methylation on arginine is identified but the type of methylation is not clear.

Annotation	Full Name	Amino Acid Residue with the Identified Modification	Modification Subfamily	Modification Family
*as follows*	acetylation	*as follows*	acetylation	acylation
N-ac	* N*-terminal acetylation	*N*-terminus (M, S, T, P)	*as above*	*as above*
ac	* N*^ε^-acetylation	K	*as above*	*as above*
but	butyrylation	K	butyrylation	acylation
cro	crotonylation	K	crotonylation	acylation
for	formylation	K	formylation	acylation
glu	glutarylation	K	glutarylation	acylation
bhb	*β*-hydroxybutyrylation	K	*β*-hydroxybutyrylation	acylation
hib	2-hydroxyisobutyrylation	K	2-hydroxyisobutyrylation	acylation
lac	lactylation	K	lactylation	acylation
mal	malonylation	K	malonylation	acylation
pal	*S*-palmitoylation	C	palmitoylation	acylation
pro	propionylation	K	propionylation	acylation
suc	succinylation	K	succinylation	acylation
ADPr	ADP-ribosylation	K, R, S, E	ADP-ribosylation	ADP-ribosylation
PAR	polyADP-ribosylation (PARylation)	K, R, S, E	ADP-ribosylation	ADP-ribosylation
*as follows*	methylation	*as follows*	methylation	alkylation
N-me3	* N*-terminal trimethylation	*N*-terminus (G)	*as above*	*as above*
me (K)	monomethylation	K	*as above*	*as above*
me2	dimethylation	K	*as above*	*as above*
me3	trimethylation	K	*as above*	*as above*
meX (K)	mono/di/trimethylation*	K	*as above*	*as above*
Nω-me	* N*^ω^-methylation	R	*as above*	*as above*
sym/asym me2	symmetric/asymmetric dimethylation**	R	*as above*	*as above*
sym me2	symmetric dimethylation	R	*as above*	*as above*
asym me2	asymmetric dimethylation	R	*as above*	*as above*
meX (R)	mono/dimethylation***	R	*as above*	*as above*
me (Q)	monomethylation	Q	*as above*	*as above*
bio	biotinylation	K	biotinylation	biotinylation
cbm	carbamoylation	K	carbamoylation	carbamoylation
cit	citrullination	R	citrullination	citrullination
dam	deamidation	N, Q	deamidation	deamidation
dop	5-glutamyl dopaminylation	Q	5-glutamyl dopaminylation	dopaminylation
N-gly	*N*-glycosylation	N	glycosylation	glycosylation
O-GlcNAc	*O*-GlcNAcylation	S, T, Y	glycosylation	glycosylation
hyd	hydroxylation	Y	hydroxylation	hydroxylation
sum	sumoylation	K	sumoylation	Lys-Gly crosslink
ub	ubiquitination	K	ubiquitination	Lys-Gly crosslink
UFM	UFMylation (modification by ubiquitin fold modifier-1)	K	UFMylation	Lys-Gly crosslink
SNO	*S*-nitrosylation	C	nitrosylation	nitrosylation
ox	oxidation	M	oxidation	oxidation
LOX-ox	LOX-mediated oxidation	K	oxidation	oxidation
ph	phosphorylation	S, T, Y, H	phosphorylation	phosphorylation
ser	5-glutamyl serotonylation	Q	5-glutamyl serotonylation	serotonylation
crosslink_T	UV-induced Histone-DNA crosslink (base T in dsDNA oligonucleotide)	K, P	UV-induced Histone-DNA crosslink	Histone-DNA crosslink
crosslink_C	UV-induced Histone-DNA crosslink (base C in dsDNA oligonucleotide)	K	UV-induced Histone-DNA crosslink	Histone-DNA crosslink

**Table 2:** The distribution of the modification entries of different confidence levels among the histone protein families.

	Histone family	Confidence level				
		CL3	CL2	CL1a	CL1b	CL1 to CL3
Modification entry counts	H1	95	574	102	831	1602
	H2A	478	243	50	431	1202
	H2B	1126	469	242	603	2440
	H3	355	107	396	275	1133
	H4	83	37	7	108	235
	all families	2137	1430	797	2248	6612
Modification entry counts/total number of amino acid residues from all variants within a histone family	H1	0.037	0.226	0.040	0.327	0.631
	H2A	0.150	0.076	0.016	0.135	0.377
	H2B	0.400	0.166	0.086	0.214	0.866
	H3	0.287	0.086	0.320	0.222	0.915
	H4	0.413	0.184	0.035	0.537	1.169
	all families	0.214	0.143	0.080	0.225	0.662

## Abbreviations

CHHM: curated catalogue of human histone modifications
HHMD: human histone modification database
HTP: high-throughput experimental identification
LTP: low-throughput experimental identification
