# Supplementary material for: CHHM: a Manually Curated Catalogue of Human Histone Modifications Revealing Hotspot Regions and Unique Distribution Patterns
Source: Int J Biol Sci. 2024 Jul 2;20(10):3760–72. doi: 10.7150/ijbs.95954 (PMC11302869; doi:10.7150/ijbs.95954)

Supporting Information

CHHM: a Manually Curated Catalogue of Human Histone Modifications Revealing Hotspot Regions and Unique Distribution Patterns

Wendong Ma[1]*, Xiaofan Ding[1]*, Jiajia Xu[1] and Terence Chuen Wai Poon[1#]

[1] Institute of Translational Medicine, Centre for Precision Medicine Research and Training, MoE Frontiers Science Center for Precision Oncology, Faculty of Health Sciences, University of Macau, Macau, 999078, China

*These authors contributed equally to this work.

[#]Corresponding author:
Terence Chuen Wai Poon
Associate Professor
Faculty of Health Sciences, University of Macau, Macau, China
Office Tel: +853 8822-4501
Email: tcwpoon@um.edu.mo

Table of Contents

Table S1. Summary of identified human histone variants. ---------------------------------- Page S3

Table S2. The list of representative human histone variants for hotspot analysis. -- Page S7

Figure S1. The distribution patterns of various sites of modifications among human histone families H1, H2A, H2B, H3, and H4, at confidence levels of CL2 to CL3. ------------------------ Page S9

Table S1. Summary of identified human histone variants. Human histone variants include 11 variants of human H1, 21 variants of human H2A, 21 variants of human H2B, 9 variants of human H3, and 2 variants of human H4. Protein information is retrieved from UniProt, accessed in Dec 2022.

**UniProt Primary Accession**	**UniProt Accession Suffix**	**Protein Name**	**Gene Name (Synonyms Name)**	**Chromosomal Location**	**Family**
**P07305**	H10_HUMAN	Histone H1.0	*H1-0 (H1F0, H1FV)*	22q13.1	H1
**Q02539**	H11_HUMAN	Histone H1.1	*H1-1 (H1F1, HIST1H1A)*	6p22.2	H1
**P16403**	H12_HUMAN	Histone H1.2	*H1-2 (H1F2, HIST1H1C)*	6p22.2	H1
**P16402**	H13_HUMAN	Histone H1.3	*H1-3 (H1F3, HIST1H1D)*	6p22.2	H1
**P10412**	H14_HUMAN	Histone H1.4	*H1-4 (H1F4, HIST1H1E)*	6p22.2	H1
**P16401**	H15_HUMAN	Histone H1.5	*H1-5 (H1F5, HIST1H1B)*	6p22.1	H1
**P22492**	H1T_HUMAN	Histone H1t	*H1-6 (H1FT, H1T, HIST1H1T)*	6p22.2	H1
**Q75WM6**	H1FNT_HUMAN	Testis-specific H1 histone	*H1-7 (H1FNT, HANP1)*	12q13.11	H1
**Q8IZA3**	H18_HUMAN	Histone H1.8	*H1-8 (H1FOO, H1OO, OSH1)*	3q22.1	H1
**P60008**	HILS1_HUMAN	Putative spermatid-specific linker histone H1-like protein	*H1-9P (H1-9, HILS1)*	17q21.33	H1
**Q92522**	H1X_HUMAN	Histone H1.10	*H1-10 (H1FX)*	3q21.3	H1
**P0C0S8**	H2A1_HUMAN	Histone H2A type 1	*H2AC11 (H2AFP, HIST1H2AG); H2AC13 (H2AFC, HIST1H2AI); H2AC15 (H2AFD, HIST1H2AK); H2AC16 (H2AFI, HIST1H2AL); H2AC17 (H2AFN, HIST1H2AM)*	6p22.1	H2A
**Q96QV6**	H2A1A_HUMAN	Histone H2A type 1-A	*H2AC1 (H2AFR, HIST1H2AA)*	6p22.2	H2A
**P04908**	H2A1B_HUMAN	Histone H2A type 1-B/E	*H2AC4 (H2AFM, HIST1H2AB); H2AC8 (H2AFA, HIST1H2AE)*	6p22.2	H2A
**Q93077**	H2A1C_HUMAN	Histone H2A type 1-C	*H2AC6 (H2AFL, HIST1H2AC)*	6p22.2	H2A
**P20671**	H2A1D_HUMAN	Histone H2A type 1-D	*H2AC7 (H2AFG, HIST1H2AD)*	6p22.2	H2A

**Q96KK5**	H2A1H_HUMAN	Histone H2A type 1-H	*H2AC12 (HIST1H2AH, HIST1H2AI)*	6p22.1	H2A
**Q99878**	H2A1J_HUMAN	Histone H2A type 1-J	*H2AC14 (H2AFE, HIST1H2AJ)*	6p22.1	H2A
**Q6FI13**	H2A2A_HUMAN	Histone H2A type 2-A	*H2AC18 (H2AFO, HIST2H2AA, HIST2H2AA3); H2AC19 (HIST2H2AA4)*	1q21.2	H2A
**Q8IUE6**	H2A2B_HUMAN	Histone H2A type 2-B	*H2AC21 (HIST2H2AB)*	1q21.2	H2A
**Q16777**	H2A2C_HUMAN	Histone H2A type 2-C	*H2AC20 (H2AFQ, HIST2H2AC)*	1q21.2	H2A
**Q7L7L0**	H2A3_HUMAN	Histone H2A type 3	*H2AC25 (H2AW, HIST3H2A)*	1q42.13	H2A
**P0C5Y9**	H2AB1_HUMAN	Histone H2A-Bbd type 1	*H2AB1 (H2AFB1)*	Xq28	H2A
**P0C5Z0**	H2AB2_HUMAN	Histone H2A-Bbd type 2/3	*H2AB2 (H2AFB2); H2AB3 (H2ABBD, H2AFB, H2AFB3)*	Xq28	H2A
**Q9BTM1**	H2AJ_HUMAN	Histone H2A.J	*H2AJ (H2AFJ)*	12p12.3	H2A
**A0A3B3IU63**	H2AL3_HUMAN	Histone H2A-like 3	*H2AL3 (H2AL1RP)*	Xp11.4	H2A
**Q71UI9**	H2AV_HUMAN	Histone H2A.V	*H2AZ2 (H2AFV, H2AV)*	7p13	H2A
**P16104**	H2AX_HUMAN	Histone H2AX	*H2AX (H2AFX)*	11q23.3	H2A
**O75367-2**	H2AY_HUMAN	Core histone macro-H2A.1	*MACROH2A1 (H2AFY)*	5q31.1	H2A
**Q9P0M6**	H2AW_HUMAN	Core histone macro-H2A.2*	*MACROH2A2 (H2AFY2)*	10q22.1	H2A
**P0C0S5**	H2AZ_HUMAN	Histone H2A.Z	*H2AZ1 (H2AFZ, H2AZ)*	4q23	H2A
**O75409**	HYPM_HUMAN	Huntingtin-interacting protein M*	*H2AP (CXorf27, HYPM)*	Xp11.4	H2A
**Q96A08**	H2B1A_HUMAN	Histone H2B type 1-A	*H2BC1 (HIST1H2BA, TSH2B)*	6p22.2	H2B
**P33778**	H2B1B_HUMAN	Histone H2B type 1-B	*H2BC3 (H2BFF, HIST1H2BB)*	6p22.2	H2B
**P62807**	H2B1C_HUMAN	Histone H2B type 1-C/E/F/G/I	*H2BC4 (H2BFL, HIST1H2BC); H2BC6 (H2BFH, HIST1H2BE); H2BC7 (H2BFG, HIST1H2BF); H2BC8 (H2BFA, HIST1H2BG); H2BC10 (H2BFK, HIST1H2BI)*	6p22.2	H2B
**P58876**	H2B1D_HUMAN	Histone H2B type 1-D	*H2BC5 (H2BFB, HIRIP2, HIST1H2BD)*	6p22.2	H2B
**Q93079**	H2B1H_HUMAN	Histone H2B type 1-H	*H2BC9 (H2BFJ, HIST1H2BH)*	6p22.2	H2B
**P06899**	H2B1J_HUMAN	Histone H2B type 1-J	*H2BC11 (H2BFR, HIST1H2BJ)*	6p22.1	H2B

**O60814**	H2B1K_HUMAN	Histone H2B type 1-K	*H2BC12 (H2BFT, HIRIP1, HIST1H2BK)*	6p22.1	H2B
**Q99880**	H2B1L_HUMAN	Histone H2B type 1-L	*H2BC13 (H2BFC, HIST1H2BL)*	6p22.1	H2B
**Q99879**	H2B1M_HUMAN	Histone H2B type 1-M	*H2BC14 (H2BFE, HIST1H2BM)*	6p22.1	H2B
**Q99877**	H2B1N_HUMAN	Histone H2B type 1-N	*H2BC15 (H2BFD, HIST1H2BN)*	6p22.1	H2B
**P23527**	H2B1O_HUMAN	Histone H2B type 1-O	*H2BC17 (H2BFH, H2BFN, HIST1H2BO)*	6p22.1	H2B
**Q6DN03**	H2B2C_HUMAN	Putative histone H2B type 2-C	*H2BC20P (HIST2H2BC)*	1q21.2	H2B
**Q6DRA6**	H2B2D_HUMAN	Putative histone H2B type 2-D	*H2BC19P (HIST2H2BD)*	1q21.2	H2B
**Q16778**	H2B2E_HUMAN	Histone H2B type 2-E	*H2BC21 (H2BFQ, HIST2H2BE)*	1q21.2	H2B
**Q5QNW6**	H2B2F_HUMAN	Histone H2B type 2-F	*H2BC18 (HIST2H2BF)*	1q21.2	H2B
**Q8N257**	H2B3B_HUMAN	Histone H2B type 3-B	*H2BC26 (H2BU1, HIST3H2BB)*	1q42.13	H2B
**P0C1H6**	H2BFM_HUMAN	Histone H2B type F-M	*H2BW2 (H2BFM)*	Xq22.2	H2B
**P57053**	H2BFS_HUMAN	Histone H2B type F-S	*H2BC12L (H2BFS, H2BS1)*	21q22.3	H2B
**A0A2R8Y619**	H2BK1_HUMAN	Histone H2B type 2-K1	*H2BK1 (H2BE1)*	7q36.1	H2B
**P0DW85**	H2BN1_HUMAN	Histone H2A.N	*H2BN1*	17q11.2	H2B
**Q7Z2G1**	H2BWT_HUMAN	Histone H2B type W-T	*H2BW1 (H2BFWT)*	Xq22.2	H2B
**P68431**	H31_HUMAN	Histone H3.1	*H3C1 (H3FA, HIST1H3A); H3C2 (H3FL, HIST1H3B); H3C3 (H3FC HIST1H3C); H3C4 (H3FB, HIST1H3D); H3C6 (H3FD, HIST1H3E); H3C7 (H3FI, HIST1H3F); H3C8 (H3FH, HIST1H3G); H3C10 (H3FK, HIST1H3H); H3C11 (H3FF, HIST1H3I); H3C12 (H3FJ, HIST1H3J)*	6p22.2, 6p22.1 (H3C10, H3C11, H3C12)	H3
**Q71DI3**	H32_HUMAN	Histone H3.2	*H3C13 (HIST2H3D); H3C14 (H3F2, H3FM, HIST2H3C); H3C15 (HIST2H3A)*	1q21.2	H3
**P84243**	H33_HUMAN	Histone H3.3	*H3-3A (H3.3A, H3F3, H3F3A); H3-3B (H3.3B, H3F3B)*	1q42.12 (H3-3A), 17q25.1 (H3-3B)	H3
**Q16695**	H31T_HUMAN	Histone H3.1t	*H3-4 (H3FT, HIST3H3)*	1q42.13	H3

**Q6NXT2**	H3C_HUMAN	Histone H3.3C	*H3-5 (H3F3C)*	12p11.21	H3
**Q5TEC6**	H37_HUMAN	Histone H3-7	*H3-7 (H3-2, HIST2H3PS2)*	1q21.1	H3
**P0DPK2**	H3Y1_HUMAN	Histone H3.Y	*H3Y1*	5p15.1	H3
**P0DPK5**	H3Y2_HUMAN	Histone H3.X	*H3Y2*	5p15.1	H3
**P49450**	CENPA_HUMAN	Histone H3-like centromeric protein A	*CENPA*	2p23.3	H3
**P62805**	H4_HUMAN	Histone H4	*H4C1 (H4/A, H4FA, HIST1H4A); H4C2 (H4/I, H4FI, HIST1H4B); H4C3 (H4/G, H4FG, HIST1H4C); H4C4 (H4/B, H4FB, HIST1H4D); H4C5 (H4/J, H4FJ, HIST1H4E); H4C6 (H4/C, H4FC, HIST1H4F); H4C8 (H4/H, H4FH, HIST1H4H); H4C9 (H4/M, H4FM, HIST1H4I); H4C11 (H4/E, H4FE, HIST1H4J); H4C12 (H4/D, H4FD, HIST1H4K); H4C13 (H4/K, H4FK, HIST1H4L); H4C14 (H4/N, H4F2, H4FN, HIST2H4, HIST2H4A); H4C15 (H4/O, H4FO, HIST2H4B); H4C16 (H4-16, HIST4H4)*	6p22.2, 6p22.1 (H4C9, H4C11, H4C12, H4C13), 1q21.2 (H4C14, H4C15), 12p12.3 (H4C16)	H4
**Q99525**	H4G_HUMAN	Histone H4-like protein type G	*H4C7 (H4/L, H4FL, HIST1H4G)*	6p22.2	H4

* Q9P0M6|H2AW_HUMAN and O75409|HYPM_HUMAN are not counted into H2A family in UniProt.

Table S2. The list of representative human histone variants for hotspot analysis. The representative variants refer to variants with high homology, consisting of 5 variants in H1, 12 variants in H2A, 15 variants in H2B, 7 variants in H3, and 2 (all) variants in H4.

**UniProt Primary Accession**	**Protein Name**	**Family**	**Number of Amino Acids**
Q02539	Histone H1.1	H1	215
P16403	Histone H1.2	H1	213
P16402	Histone H1.3	H1	221
P10412	Histone H1.4	H1	219
P16401	Histone H1.5	H1	226
P0C0S8	Histone H2A type 1	H2A	130
Q96QV6	Histone H2A type 1-A	H2A	131
P04908	Histone H2A type 1-B/E	H2A	130
Q93077	Histone H2A type 1-C	H2A	130
P20671	Histone H2A type 1-D	H2A	130
Q96KK5	Histone H2A type 1-H	H2A	128
Q99878	Histone H2A type 1-J	H2A	128
Q6FI13	Histone H2A type 2-A	H2A	130
Q8IUE6	Histone H2A type 2-B	H2A	130
Q16777	Histone H2A type 2-C	H2A	129
Q7L7L0	Histone H2A type 3	H2A	130
Q9BTM1	Histone H2A.J	H2A	129
Q96A08	Histone H2B type 1-A	H2B	127
P33778	Histone H2B type 1-B	H2B	126
P62807	Histone H2B type 1-C/E/F/G/I	H2B	126
P58876	Histone H2B type 1-D	H2B	126
Q93079	Histone H2B type 1-H	H2B	126
P06899	Histone H2B type 1-J	H2B	126
O60814	Histone H2B type 1-K	H2B	126
Q99880	Histone H2B type 1-L	H2B	126
Q99879	Histone H2B type 1-M	H2B	126
Q99877	Histone H2B type 1-N	H2B	126
P23527	Histone H2B type 1-O	H2B	126
Q16778	Histone H2B type 2-E	H2B	126
Q5QNW6	Histone H2B type 2-F	H2B	126
Q8N257	Histone H2B type 3-B	H2B	126
P57053	Histone H2B type F-S	H2B	126
P68431	Histone H3.1	H3	136
Q71DI3	Histone H3.2	H3	136
P84243	Histone H3.3	H3	136
Q16695	Histone H3.1t	H3	136
Q6NXT2	Histone H3.3C	H3	135

Q5TEC6	Histone H3-7	H3	136
P0DPK2	Histone H3.Y	H3	136
P62805	Histone H4	H4	103
Q99525	Histone H4-like protein type G	H4	98

Figure S1. The prevalence of various amino acid residues with modifications in different human histone families H1, H2A, H2B, H3, and H4, at confidence levels of CL2 to CL3. K, R, S, T, Y, M, C, Q, E, N, P, and Nt refer to lysine, arginine, serine, threonine, tyrosine, methionine, cysteine, glutamine, glutaric acid, asparagine, proline, and protein *N*-terminus, respectively.

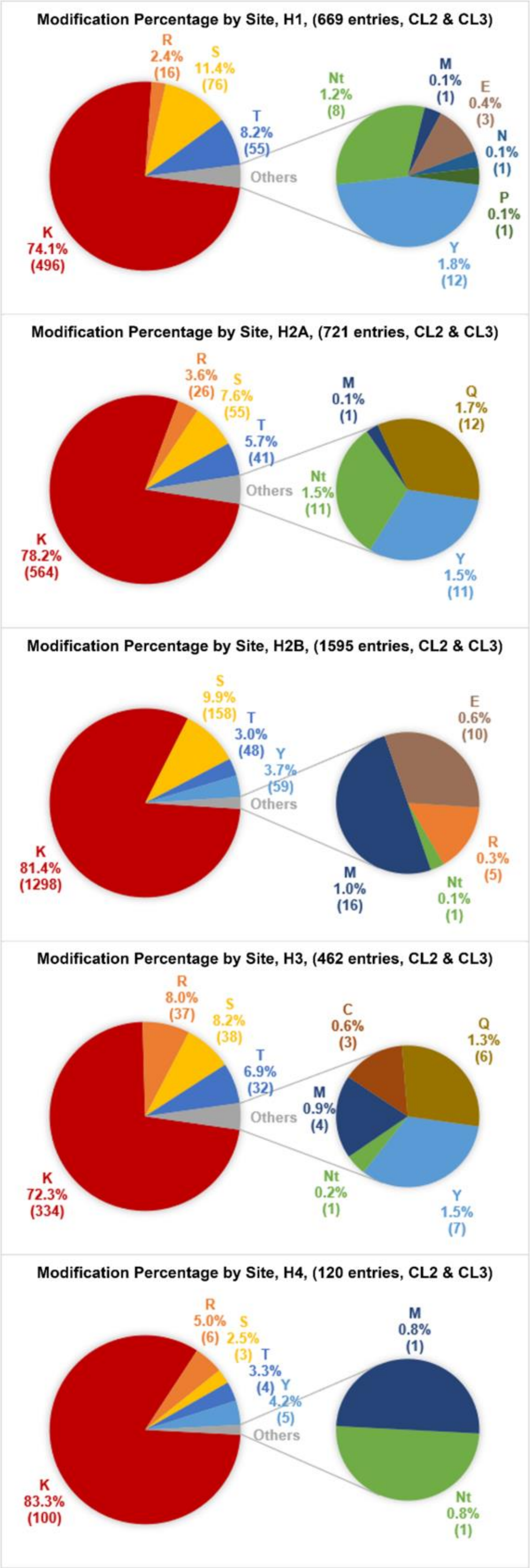

Supplement: Supplementary file 1 — Table S1. Summary of identified human histone variants. Table S2. The list of representative human histone variants for hotspot analysis. Figure S1. The distribution patterns of various sites of modifications among human histone families H1, H2A, H2B, H3, and H4, at confidence levels of CL2 to CL3, (PDF). [file ijbsv20p3760s1.pdf]
