# Supplementary material for: CHHM: a Manually Curated Catalogue of Human Histone Modifications Revealing Hotspot Regions and Unique Distribution Patterns
Source: Int J Biol Sci. 2024 Jul 2;20(10):3760–72. doi: 10.7150/ijbs.95954 (PMC11302869; doi:10.7150/ijbs.95954)

User Manual

Curated Catalogue of Human Histone Modifications (CHHM)

Table of Contents

1. Introduction of CHHM ……………………………………… 3

2. Structure of CHHM ………………………………………… 8

3. How to use CHHM ………………………………………… 19

3A. Scenery A ………………………………………… 21

3B. Scenery B ………………………………………… 27

1. Introduction of CHHM

Welcome to the Curated Catalogue of Human Histone Modifications (CHHM)!

CHHM is a tool for convenient viewing and locating human histone modifications, obtained by manual curation of modification records from 10 knowledgebases/databases and 3 complementary articles.

CHHM contains 6612 nonredundant modification entries covering 31 types of modifications (including 9 types of emerging modifications) and 2 types of histone-DNA crosslinks, that were identified in 11 H1 variants, 21 H2A variants, 21 H2B variants, 9 H3 variants, and 2 H4 variants. Among the 6612 nonredundant modification entries, 4364 entries were retrieved from knowledgebases/databases and complementary articles, 2248 were identified by inference based on protein sequence similarity and only identified in the present study.

Modification entries are presented with aligned protein sequences for ease of visualization and accessibility. All retrieved modification records were manually inspected and classified into 3 categories based on the confidence level according to experimental evidence.

This tool is contributed and published by Wendong MA, Xiaofan DING, Jiajia XU and Terence Chuen Wai POON (Corresponding Author), Faculty of Health Sciences (FHS), University of Macau

Website of Prof. Terence Chuen Wai POON: https://fhs.um.edu.mo/en/staff/terence-chuen-wai-poon/
Website of Prof. Xiaofan DING: https://fhs.um.edu.mo/en/staff/xiaofan-ding/
Website of Faculty of Health Sciences (FHS): https://fhs.um.edu.mo/
Website of University of Macau: https://www.um.edu.mo/

KNOWLEDGEBASE/DATABASE INFO

Through literature search, 16 publicly accessible knowledgebases/databases containing records of human histone modifications were identified. After manual comparison of sources of the modification records among the knowledgebases/databases, 10 publicly accessible knowledgebases/databases were included in the present work (as follows), 6 knowledgebases/databases (Phosida, IntAct, PRO, HPRD, neXtProt, Signor) were excluded to avoid redundancy. Each included knowledgebase/database was annotated with a unique capital letter in our curated modification catalogue.

**Knowledgebase/Database**	**Annotation**	**Type of Histone Modifications**	**Experimental Evidence of Provided Information**	**Whether Providing Inference by Similarity**	**Whether Classifying Subtypes of Methylation**	**Primary Source(s) of Modification Records**
iPTMnet	*N*	Many	LTP/HTP/ISD	N	N	UniProtKB, PhosphoSitePlus, phospho.ELM, GlyGen, dbSNO, HPRD, IntAct, PRO, BioGRID, Biomuta, neXtProt, p3DB, PhosPhAt, PomBase, RLIMS-P, SGD, Signor, etc.
UniProtKB (Swiss-Prot)	*U*	Many	LTP/HTP/ISD	Y	Y	PRIDE
PhosphoSitePlus	*P*	Many	LTP/HTP/ISD	Y	Y	-
Phospho.ELM	*E*	Phosphorylation	LTP/HTP	N	N	PhosphoSitePlus, Phosida
HISTome2	*H*	Many	LTP	N	Y	-
GlyGen	*G*	Glycosylation	LTP/ISD	Y	N	O-GlcNAc Database, iPTMnet, UniProtKB, phospho.ELM, PRO, etc.
GlyConnect	*L*	Glycosylation	LTP	N	N	-
MetOSite	*M*	Methionine Oxidation	LTP	N	N	PhosphoSitePlus
SwissPalm	*S*	Palmitoylation	LTP	N	N	-
dbSNO	*D*	Nitrosylation	LTP/HTP	N	N	UniProtKB, PhosphoSitePlus, Phospho.ELM, OGlycBase, dbPTM 3.0, dbGSH, RegPhos 2.0, KinasePhos, etc.

ARTICLE INFO

Three articles were also included (as follows), because they provided complementary records of human histone modifications which have not been included by the 10 knowledgebases/databases.

Article	Symbol	Introduction
Stützer et al., *Nat. Commun*., 2020	1	A novel and unique article for the first time identified sites for UV-induced histone-DNA crosslinks on histone H1.4. These crosslinks are not collected by the 10 knowledgebases/databases.
O’Neil et al., *Sci Adv*., 2020	2	A representative article identifying calbamoylation sites on histones. Carbamylation is not collected by the 10 knowledgebases/databases.
Andrés et al., *Int J Mol Sci*., 2020	3	A representative review systematically discussing modifications on Histone family H1. Formylation and hydroxylation are not collected by the 10 knowledgebases/databases.

For detailed information retrieved from articles, please refer to the last sheet "Entries featured in articles".

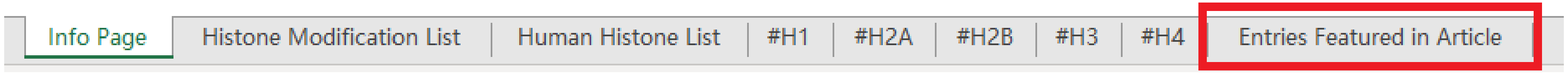

CONFIDENCE LEVEL

All retrieved modification records were manually inspected and classified into 3 categories based on the confidence level according to experimental evidence.

The highest level of confidence (CL3) was given to those modifications identified/confirmed by low-throughout experimental methods, i.e., typical experiments generating solid evidence. A middle level of confidence (CL2) was given to those only identified by high-throughput experimental methods, e.g., mass spectrometry-based bottom-up proteomic experiments. The lowest level of confidence (CL1, including CL1a and CL1b) was given to those modifications identified by inference based on protein sequence similarity.

These confidence levels of the modification records were defined as follows:

Confidence Level	Experimental Evidence of Records
CL3	Generated by low-throughput experimental identification (LTP).
CL2	Only generated by high-throughput experimental identification (HTP).
CL1a	Identified by inference based on protein sequence similarity and recorded in at least one of the 10 knowledgebases/databases (ISD).
CL1b	Identified by inference based on protein sequence similarity and only identified in the present study (ISS).

2. Structure of CHHM

The tool CHHM consists of nine worksheets:

Worksheet 1: Information page
Worksheet 2: List of histone modifications and crosslinks included in CHHM
Worksheet 3: List of human histone variants
Worksheets 4-8: Curated modification entries in human histone families
Worksheet 9: List of modification entries featured in three articles

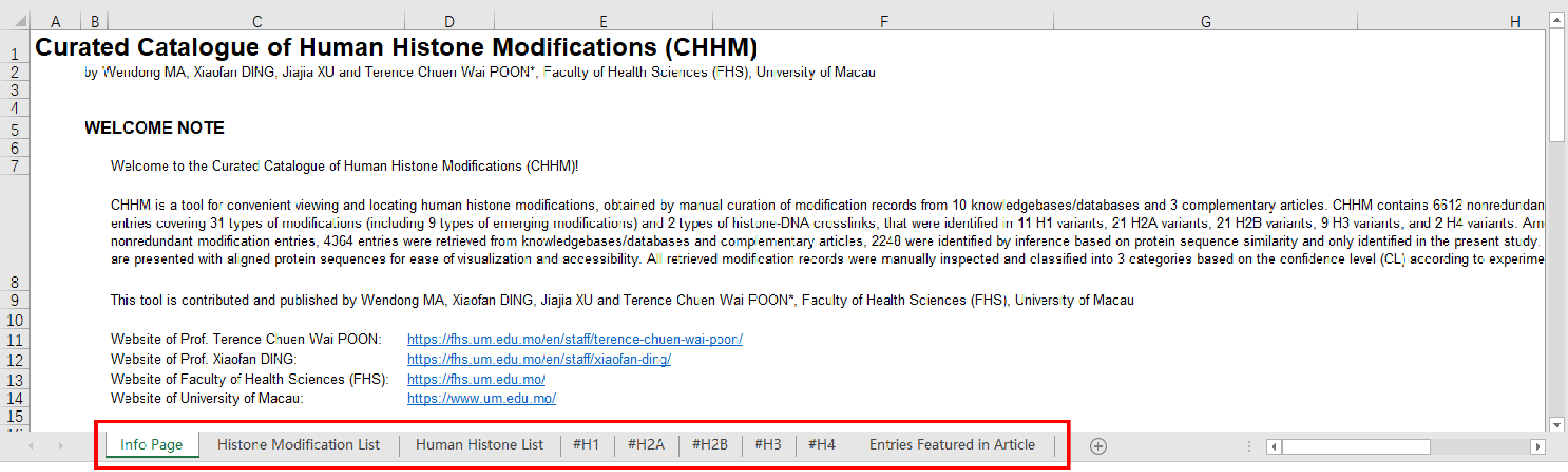

Curated Catalogue of Human Histone Modifications (CHHM)

by Wendong MA, Xiaofan DING, Jiajia XU and Terence Chuen Wai POON*, Faculty of Health Sciences (FHS), University of Macau

WELCOME NOTE

Welcome to the Curated Catalogue of Human Histone Modifications (CHHM)!

CHHM is a tool for convenient viewing and locating human histone modifications, obtained by manual curation of modification records from 10 knowledgebases/databases and 3 complementary articles. CHHM contains 6612 nonredundan entries covering 31 types of modifications (including 9 types of emerging modifications) and 2 types of histone-DNA crosslinks, that were identified in 11 H1 variants, 21 H2A variants, 21 H2B variants, 9 H3 variants, and 2 H4 variants. Am nonredundant modification entries, 4364 entries were retrieved from knowledgebases/databases and complementary articles, 2248 were identified by inference based on protein sequence similarity and only identified in the present study. are presented with aligned protein sequences for ease of visualization and accessibility. All retrieved modification records were manually inspected and classified into 3 categories based on the confidence level (CL) according to experime

This tool is contributed and published by Wendong MA, Xiaofan DING, Jiajia XU and Terence Chuen Wai POON*, Faculty of Health Sciences (FHS), University of Macau

Website of Prof. Terence Chuen Wai POON: https://fhs.um.edu.mo/en/staff/terence-chuen-wai-poon/
Website of Prof. Xiaofan DING: https://fhs.um.edu.mo/en/staff/xiaofan-ding/
Website of Faculty of Health Sciences (FHS): https://fhs.um.edu.mo/
Website of University of Macau: https://www.um.edu.mo/

Info Page | Histone Modification List | Human Histone List | #H1 | #H2A | #H2B | #H3 | #H4 | Entries Featured in Article

Worksheet 1: Information page

Information page provides a fundamental introduction of the tool CHHM, the information of knowledgebases/databases and articles retrieved in this tool, and classification of confidence levels.

A	B	C	D	E	F	G	H

Curated Catalogue of Human Histone Modifications (CHHM)

by Wendong MA, Xiaofan DING, Jiajia XU and Terence Chuen Wai POON*, Faculty of Health Sciences (FHS), University of Macau

WELCOME NOTE

Welcome to the Curated Catalogue of Human Histone Modifications (CHHM)!

CHHM is a tool for convenient viewing and locating human histone modifications, obtained by manual curation of modification records from 10 knowledgebases/databases and 3 complementary articles. CHHM contains 6612 nonredundan entries covering 31 types of modifications (including 9 types of emerging modifications) and 2 types of histone-DNA crosslinks, that were identified in 11 H1 variants, 21 H2A variants, 21 H2B variants, 9 H3 variants, and 2 H4 variants. Am nonredundant modification entries, 4364 entries were retrieved from knowledgebases/databases and complementary articles, 2248 were identified by inference based on protein sequence similarity and only identified in the present study. are presented with aligned protein sequences for ease of visualization and accessibility. All retrieved modification records were manually inspected and classified into 3 categories based on the confidence level (CL) according to experime

This tool is contributed and published by Wendong MA, Xiaofan DING, Jiajia XU and Terence Chuen Wai POON*, Faculty of Health Sciences (FHS), University of Macau

Website of Prof. Terence Chuen Wai POON: https://fhs.um.edu.mo/en/staff/terence-chuen-wai-poon/
Website of Prof. Xiaofan DING: https://fhs.um.edu.mo/en/staff/xiaofan-ding/
Website of Faculty of Health Sciences (FHS): https://fhs.um.edu.mo/
Website of University of Macau: https://www.um.edu.mo/

Info Page | Histone Modification List | Human Histone List | #H1 | #H2A | #H2B | #H3 | #H4 | Entries Featured in Article

Worksheet 2: List of histone modifications and crosslinks included in CHHM

This worksheet provides summary of 31 types of modifications and 2 types of crosslinks included in CHHM. For acetylation and methylation, annotations of their subtypes are presented. This part of information is consistent to Table 1 in the manuscript.

	A	B	C	D	E
1	**Annotation**	**Full Name**	**Amino Acid Residue with the Identified Modification**	**Modification Subfamily**	**Modification Family**
2	*as follows*	acetylation	*as follows*	acetylation	acylation
3	N-ac	*N*-terminal acetylation	*N*-terminus (M, S, T, P)	*as above*	*as above*
4	ac	N^{ε}-acetylation	K	*as above*	*as above*
5	but	butyrylation	K	butyrylation	acylation
6	cro	crotonylation	K	crotonylation	acylation
7	for	formylation	K	formylation	acylation
8	glu	glutarylation	K	glutarylation	acylation
9	bhb	*β*-hydroxybutyrylation	K	*β*-hydroxybutyrylation	acylation
10	hib	2-hydroxyisobutyrylation	K	2-hydroxyisobutyrylation	acylation
11	lac	lactylation	K	lactylation	acylation
12	mal	malonylation	K	malonylation	acylation
13	pal	*S*-palmitoylation	C	palmitoylation	acylation
14	pro	propionylation	K	propionylation	acylation
15	suc	succinylation	K	succinylation	acylation
16	ADPr	ADP-ribosylation	K, R, S, E	ADP-ribosylation	ADP-ribosylation
17	PAR	polyADP-ribosylation (PARylation)	K, R, S, E	ADP-ribosylation	ADP-ribosylation
18	*as follows*	methylation	*as follows*	methylation	alkylation
19	N-me3	*N*-terminal trimethylation	*N*-terminus (G)	*as above*	*as above*
20	me (K)	monomethylation	K	*as above*	*as above*
21	me2	dimethylation	K	*as above*	*as above*
22	me3	trimethylation	K	*as above*	*as above*

Info Page | Histone Modification List | Human Histone List | #H1 | #H2A | #H2B | #H3 | #H4 | Entries Featured in Article

Worksheet 3: List of human histone variants

This worksheet provides summary of identified human histone variants. Human histone variants include 11 variants of human H1, 21 variants of human H2A, 21 variants of human H2B, 9 variants of human H3, and 2 variants of human H4. Protein information is retrieved from UniProt, accessed in Dec 2022. This part of information is consistent to Table S1 in the supporting information.

	A	B	C	D	
1	UniProt Primary Accession	UniProt Secondary Accession	UniProt Accession Suffix	Protein Name	Short
2	P07305	B2R6I0; B4DRD6; Q6FG88; Q8N6R3	H10_HUMAN	Histone H1.0	Histor
3	Q02539	Q3MJ34	H11_HUMAN	Histone H1.1	Histor
4	P16403	A8K4I2	H12_HUMAN	Histone H1.2	Histor
5	P16402	B2R751; Q2M2I2	H13_HUMAN	Histone H1.3	Histor
6	P10412	Q4VB25	H14_HUMAN	Histone H1.4	Histor
7	P16401	Q14529; Q3MJ42	H15_HUMAN	Histone H1.5	Histor
8	P22492	Q6ISI1; Q8IUE8	H1T_HUMAN	Histone H1t	Testic
9	Q75WM6	Q147U8; Q5GKZ5; Q7Z694	H1FNT_HUMAN	Testis-specific H1 histone	Haploi
10	Q8IZA3	Q86WT7	H18_HUMAN	Histone H1.8	Histor
11	P60008	-	HILS1_HUMAN	Putative spermatid-specific linker histone H1-like protein	H1.9 l
12	Q92522	-	H1X_HUMAN	Histone H1.10	Histor
13	P0C0S8	P02261; Q2M1R2; Q76PA6	H2A1_HUMAN	Histone H2A type 1	H2A.1
14	Q96QV6	-	H2A1A_HUMAN	Histone H2A type 1-A	H2A-c
15	P04908	P28001; Q76P63	H2A1B_HUMAN	Histone H2A type 1-B/E	Histor
16	Q93077	B2R4F7; O00775; O00776; O00777; O00778; Q540R1	H2A1C_HUMAN	Histone H2A type 1-C	H2A-c
17	P20671	A0PK91; P57754; Q6FGY6	H2A1D_HUMAN	Histone H2A type 1-D	Histor
18	Q96KK5	-	H2A1H_HUMAN	Histone H2A type 1-H	H2A-c
19	Q99878	A2RUU6; Q5JXQ5	H2A1J_HUMAN	Histone H2A type 1-J	Histor
20	Q6FI13	B2R5F0; P20670	H2A2A_HUMAN	Histone H2A type 2-A	H2A-c
21	Q8IUE6	-	H2A2B_HUMAN	Histone H2A type 2-B	H2A-c
22	Q16777	Q6DRA7; Q8IUE5	H2A2C_HUMAN	Histone H2A type 2-C	H2A-c
[illegible]	[illegible]	[illegible]	[illegible]	[illegible]	[illegible]

Info Page | Histone Modification List | Human Histone List | #H1 | #H2A | #H2B | #H3 | #H4 | Entries Featured in Article

Worksheets 4-8: Curated modification entries in human histone families (Part 1)

Worksheets 4-8 are the principal content in CHHM, providing the curated modification entries in human histone families H1, H2A, H2B, H3, and H4, respectively. Below is the key table for these worksheets. The curated modification entries in CHHM are delivered at two levels: protein sequence level and modification level. At protein sequence level, capital letters denote amino acid symbols (e.g., M for methionine, K for lysine). At modification level, capital letters or numbers in italic denote the source of a modification entry (e.g., *N* for a modification featured in iPTMnet; *2* for a modification featured in O'Neil et al., *Sci adv.*, 2020). At both protein sequence level and modification level, font color and background color denote the confidence level as shown in the key table. Besides, the initial methionine is indicated with a red background color. This part of information is consistent to Figure 1 in manuscript.

	KEYS
	**PROTEIN SEQUENCE LEVEL**
M	Initiator Methionine
K	Site information supported by LTP [Confidence Level 3]
K	Site information only supported by HTP [Confidence Level 2]
K	Site information supported by ISD [Confidence Level 1a]
K	Site information supported by ISS [Confidence Level 1b]
	**MODIFICATION LEVEL**
N	Modification featured in iPTMnet
U	Modification featured in UniProtKB
P	Modification featured in PhosphoSitePlus
E	Modification featured in Phospho.ELM
H	Modification featured in the HISTome2
G	Modification featured in GlyGen (Glycosylation)
L	Modification featured in GlyConnect (Glycosylation)
M	Modification featured in MetOSite (Methionine Oxidation)
S	Modification featured in SwissPalm (Palmitoylation)
D	Modification featured in dbSNO (S-nitrosylation)
1	Modification featured in article (Stützer et al., *Nat. Commun.* , 2020) (UV-induced histone-DNA crosslinks in H1 family)
2	Modification featured in article (O'Neil et al., *Sci Adv.* , 2020) (Carbamoylation in H3 and H4 families)
3	Modification featured in article (Andrés et al., *Int J Mol Sci.* , 2020) (H1 modifications)
U	Modification record generated by LTP, featured in the corresponding knowledgebase/database or article
U	Modification record only generated by HTP, featured in the corresponding knowledgebase/database or article
U	Modification record generated by ISD, featured in the corresponding knowledgebase/database
i	Modification record generated by ISS

Worksheets 4-8: Curated modification entries in human histone families (Part 2)

The next page demonstrates an example page of CHHM providing information of the modification entries at protein sequence level.

At protein sequence level, aligned amino acid sequences of the histone variants within a family are provided. Gaps in protein sequence are determined by sequence alignment. The confidence of the amino acid residue being a modification site is indicated by the confidence level of the modification entry with the highest confidence of evidence among all entries associated with the amino acid residue. The degree of confidence is denoted by a combination of the background color and the color of the amino acid symbol (refer to the key table for details). The initial methionine is highlighted with a red background color.

This part of information is consistent to Figure 2 in manuscript.

Protein accession

Initial methionine

Unmodified site

Modified site

Gap determined by sequence alignment

Protein sequence position of an amino acid residue presented at the end of a line

	A	B	C	D	E	F	G	H	I	J	K	L	M	N	O	P	Q	R	S	T	U	V	W	X	Y	Z	AA	AB	AC	AD	AE	AF	AG	AH	AI	AJ	AK	AL	AM	AN	AO	AP	AQ	AR	AS	AT	AU	AV	AW	AX	AY	AZ	BA	BB	BC	BD	BE	BF	BG	BH	BI	BJ	BK
31																																																																
32	**UniProt Accession**																																																															
33	P0C0S8	H2A1_HUMAN	-	-	-	-	M	S	G	R	G	K	Q	G	G	K	A	R	A	K	A	K	T	R	S	S	R	A	G	L	Q	F	P	V	G	R	V	H	R	L	L	R	K	G	-	N	Y	A	E	R	V	G	A	G	A	P	V	Y	L	A	A	V	55
50	Q96QV6	H2A1A_HUMAN	-	-	-	-	M	S	G	R	G	K	Q	G	G	K	A	R	A	K	S	K	S	R	S	S	R	A	G	L	Q	F	P	V	G	R	I	H	R	L	L	R	K	G	-	N	Y	A	E	R	I	G	A	G	A	P	V	Y	L	A	A	V	55
67	P04908	H2A1B_HUMAN	-	-	-	-	M	S	G	R	G	K	Q	G	G	K	A	R	A	K	A	K	T	R	S	S	R	A	G	L	Q	F	P	V	G	R	V	H	R	L	L	R	K	G	-	N	Y	S	E	R	V	G	A	G	A	P	V	Y	L	A	A	V	55
84	Q93077	H2A1C_HUMAN	-	-	-	-	M	S	G	R	G	K	Q	G	G	K	A	R	A	K	A	K	S	R	S	S	R	A	G	L	Q	F	P	V	G	R	V	H	R	L	L	R	K	G	-	N	Y	A	E	R	V	G	A	G	A	P	V	Y	L	A	A	V	55
10	P20671	H2A1D_HUMAN	-	-	-	-	M	S	G	R	G	K	Q	G	G	K	A	R	A	K	A	K	T	R	S	S	R	A	G	L	Q	F	P	V	G	R	V	H	R	L	L	R	K	G	-	N	Y	S	E	R	V	G	A	G	A	P	V	Y	L	A	A	V	55
11	Q96KK5	H2A1H_HUMAN	-	-	-	-	M	S	G	R	G	K	Q	G	G	K	A	R	A	K	A	K	T	R	S	S	R	A	G	L	Q	F	P	V	G	R	V	H	R	L	L	R	K	G	-	N	Y	A	E	R	V	G	A	G	A	P	V	Y	L	A	A	V	55
13	Q99878	H2A1J_HUMAN	-	-	-	-	M	S	G	R	G	K	Q	G	G	K	A	R	A	K	A	K	T	R	S	S	R	A	G	L	Q	F	P	V	G	R	V	H	R	L	L	R	K	G	-	N	Y	A	E	R	V	G	A	G	A	P	V	Y	L	A	A	V	55
15	Q6FI13	H2A2A_HUMAN	-	-	-	-	M	S	G	R	G	K	Q	G	G	K	A	R	A	K	A	K	S	R	S	S	R	A	G	L	Q	F	P	V	G	R	V	H	R	L	L	R	K	G	-	N	Y	A	E	R	V	G	A	G	A	P	V	Y	M	A	A	V	55
17	Q8IUE6	H2A2B_HUMAN	-	-	-	-	M	S	G	R	G	K	Q	G	G	K	A	R	A	K	A	K	S	R	S	S	R	A	G	L	Q	F	P	V	G	R	V	H	R	L	L	R	K	G	-	N	Y	A	E	R	V	G	A	G	A	P	V	Y	L	A	A	V	55
18	Q16777	H2A2C_HUMAN	-	-	-	-	M	S	G	R	G	K	Q	G	G	K	A	R	A	K	A	K	S	R	S	S	R	A	G	L	Q	F	P	V	G	R	V	H	R	L	L	R	K	G	-	N	Y	A	E	R	V	G	A	G	A	P	V	Y	M	A	A	V	55
20	Q7L7L0	H2A3_HUMAN	-	-	-	-	M	S	G	R	G	K	Q	G	G	K	A	R	A	K	A	K	S	R	S	S	R	A	G	L	Q	F	P	V	G	R	V	H	R	L	L	R	K	G	-	N	Y	S	E	R	V	G	A	G	A	P	V	Y	L	A	A	V	55
22	Q9BTM1	H2AJ_HUMAN	-	-	-	-	M	S	G	R	G	K	Q	G	G	K	V	R	A	K	A	K	S	R	S	S	R	A	G	L	Q	F	P	V	G	R	V	H	R	L	L	R	K	G	-	N	Y	A	E	R	V	G	A	G	A	P	V	Y	L	A	A	V	55
23	P16104	H2AX_HUMAN	-	-	-	-	M	S	G	R	G	K	T	G	G	K	A	R	A	K	A	K	S	R	S	S	R	A	G	L	Q	F	P	V	G	R	V	H	R	L	L	R	K	G	-	H	Y	A	E	R	V	G	A	G	A	P	V	Y	L	A	A	V	55
25	O75367-2	H2AY_HUMAN	-	-	-	-	M	S	S	R	-	-	-	G	G	K	K	K	S	T	K	T	S	R	S	A	K	A	G	V	I	F	P	V	G	R	M	L	R	Y	I	K	K	G	-	H	P	K	Y	R	I	G	V	G	A	P	V	Y	M	A	A	V	52
27	Q9P0M6	H2AW_HUMAN	-	-	-	-	M	S	G	R	-	-	-	S	G	K	K	K	M	S	K	L	S	R	S	A	R	A	G	V	I	F	P	V	G	R	L	M	R	Y	L	K	K	G	-	T	F	K	Y	R	I	S	V	G	A	P	V	Y	M	A	A	V	52
29	P0C5Y9	H2AB1_HUMAN	M	P	R	R	R	R	R	R	G	S	S	G	A	G	G	R	G	R	T	C	S	R	T	V	R	A	E	L	S	F	S	V	S	Q	V	E	R	S	L	R	E	G	-	H	Y	A	Q	R	L	S	R	T	A	P	V	Y	L	A	A	V	59
29	P0C5Z0	H2AB2_HUMAN	M	P	R	R	R	R	R	R	G	S	S	G	A	G	G	R	G	R	T	C	S	R	T	V	R	A	E	L	S	F	S	V	S	Q	V	E	R	S	L	R	E	G	-	H	Y	A	Q	R	L	S	R	T	A	P	V	Y	L	A	A	V	59
30	A0A3B3IU63	H2AL3_HUMAN	-	-	M	A	G	N	K	M	F	C	R	-	-	P	R	R	Q	R	L	-	S	H	S	R	R	A	E	L	Q	F	P	V	S	H	L	E	R	C	L	R	E	S	Q	H	-	A	R	H	L	S	S	T	T	P	V	F	L	A	G	V	54
30	Q71UI9	H2AV_HUMAN	-	-	M	A	G	G	K	A	G	K	D	S	G	K	A	K	A	K	A	V	S	R	S	Q	R	A	G	L	Q	F	P	V	G	R	I	H	R	H	L	K	T	R	T	T	S	H	G	R	V	G	A	T	A	A	V	Y	S	A	A	I	58
31	P0C0S5	H2AZ_HUMAN	-	-	M	A	G	G	K	A	G	K	D	S	G	K	A	K	T	K	A	V	S	R	S	Q	R	A	G	L	Q	F	P	V	G	R	I	H	R	H	L	K	S	R	T	T	S	H	G	R	V	G	A	T	A	A	V	Y	S	A	A	I	58
33	O75409	HYPM_HUMAN	-	-	M	S	E	K	K	N	C	K	-	N	S	S	T	N	N	N	Q	T	Q	D	P	S	R	N	E	L	Q	V	P	R	S	F	V	D	R	V	V	Q	D	E	R	D	V	Q	S	Q	S	S	S	T	I	N	-	T	L	L	T	L	56
336																																																																
337	**UniProt Accession**																																																															
338	P0C0S8	H2A1_HUMAN	L	E	Y	L	T	A	E	I	L	E	L	A	G	N	A	A	R	D	N	K	K	T	R	I	I	P	R	H	L	Q	L	A	I	R	N	D	E	E	L	N	K	L	L	G	K	V	T	I	A	Q	G	G	V	L	P	N	I	Q	A	V	115
353	Q96QV6	H2A1A_HUMAN	L	E	Y	L	T	A	E	I	L	E	L	A	G	N	A	S	R	D	N	K	K	T	R	I	I	P	R	H	L	Q	L	A	I	R	N	D	E	E	L	N	K	L	L	G	G	V	T	I	A	Q	G	G	V	L	P	N	I	Q	A	V	115
366	P04908	H2A1B_HUMAN	L	E	Y	L	T	A	E	I	L	E	L	A	G	N	A	A	R	D	N	K	K	T	R	I	I	P	R	H	L	Q	L	A	I	R	N	D	E	E	L	N	K	L	L	G	R	V	T	I	A	Q	G	G	V	L	P	N	I	Q	A	V	115
379	Q93077	H2A1C_HUMAN	L	E	Y	L	T	A	E	I	L	E	L	A	G	N	A	A	R	D	N	K	K	T	R	I	I	P	R	H	L	Q	L	A	I	R	N	D	E	E	L	N	K	L	L	G	R	V	T	I	A	Q	G	G	V	L	P	N	I	Q	A	V	115
392	P20671	H2A1D_HUMAN	L	E	Y	L	T	A	E	I	L	E	L	A	G	N	A	A	R	D	N	K	K	T	R	I	I	P	R	H	L	Q	L	A	I	R	N	D	E	E	L	N	K	L	L	G	K	V	T	I	A	Q	G	G	V	L	P	N	I	Q	A	V	115
407	Q96KK5	H2A1H_HUMAN	L	E	Y	L	T	A	E	I	L	E	L	A	G	N	A	A	R	D	N	K	K	T	R	I	I	P	R	H	L	Q	L	A	I	R	N	D	E	E	L	N	K	L	L	G	K	V	T	I	A	Q	G	G	V	L	P	N	I	Q	A	V	115
422	Q99878	H2A1J_HUMAN	L	E	Y	L	T	A	E	I	L	E	L	A	G	N	A	A	R	D	N	K	K	T	R	I	I	P	R	H	L	Q	L	A	I	R	N	D	E	E	L	N	K	L	L	G	K	V	T	I	A	Q	G	G	V	L	P	N	I	Q	A	V	115
437	Q6FI13	H2A2A_HUMAN	L	E	Y	L	T	A	E	I	L	E	L	A	G	N	A	A	R	D	N	K	K	T	R	I	I	P	R	H	L	Q	L	A	I	R	N	D	E	E	L	N	K	L	L	G	K	V	T	I	A	Q	G	G	V	L	P	N	I	Q	A	V	115
452	Q8IUE6	H2A2B_HUMAN	L	E	Y	L	T	A	E	I	L	E	L	A	G	N	A	A	R	D	N	K	K	T	R	I	I	P	R	H	L	Q	L	A	V	R	N	D	E	E	L	N	K	L	L	G	G	V	T	I	A	Q	G	G	V	L	P	N	I	Q	A	V	115
465	Q16777	H2A2C_HUMAN	L	E	Y	L	T	A	E	I	L	E	L	A	G	N	A	A	R	D	N	K	K	T	R	I	I	P	R	H	L	Q	L	A	I	R	N	D	E	E	L	N	K	L	L	G	K	V	T	I	A	Q	G	G	V	L	P	N	I	Q	A	V	115
480	Q7L7L0	H2A3_HUMAN	L	E	Y	L	T	A	E	I	L	E	L	A	G	N	A	A	R	D	N	K	K	T	R	I	I	P	R	H	L	Q	L	A	I	R	N	D	E	E	L	N	K	L	L	G	R	V	T	I	A	Q	G	G	V	L	P	N	I	Q	A	V	115
493	Q9BTM1	H2AJ_HUMAN	L	E	Y	L	T	A	E	I	L	E	L	A	G	N	A	A	R	D	N	K	K	T	R	I	I	P	R	H	L	Q	L	A	I	R	N	D	E	E	L	N	K	L	L	G	K	V	T	I	A	Q	G	G	V	L	P	N	I	Q	A	V	115
508	P16104	H2AX_HUMAN	L	E	Y	L	T	A	E	I	L	E	L	A	G	N	A	A	R	D	N	K	K	T	R	I	I	P	R	H	L	Q	L	A	I	R	N	D	E	E	L	N	K	L	L	G	G	V	T	I	A	Q	G	G	V	L	P	N	I	Q	A	V	115
521	O75367-2	H2AY_HUMAN	L	E	Y	L	T	A	E	I	L	E	L	A	G	N	A	A	R	D	N	K	K	G	R	V	T	P	R	H	I	L	L	A	V	A	N	D	E	E	L	N	Q	L	L	K	G	V	T	I	A	S	G	G	V	L	P	N	I	H	P	E	112
528	Q9P0M6	H2AW_HUMAN	I	E	Y	L	A	A	E	I	L	E	L	A	G	N	A	A	R	D	N	K	K	A	R	I	A	P	R	H	I	L	L	A	V	A	N	D	E	E	L	N	Q	L	L	K	G	V	T	I	A	S	G	G	V	L	P	R	I	H	P	E	112
535	P0C5Y9	H2AB1_HUMAN	I	E	Y	L	T	A	K	V	P	E	L	A	G	N	E	A	Q	N	S	G	E	R	N	I	T	P	L	L	L	D	M	V	V	H	N	D	R	L	L	S	T	L	F	N	T	T	T	I	S	Q	-	-	V	A	P	G	E	D	-	-	115
539	P0C5Z0	H2AB2_HUMAN	I	E	Y	L	T	A	K	V	L	E	L	A	G	N	E	A	Q	N	S	G	E	R	N	I	T	P	L	L	L	D	M	V	V	H	N	D	R	L	L	S	T	L	F	N	T	T	T	I	S	Q	-	-	V	A	P	G	E	D	-	-	115
543	A0A3B3IU63	H2AL3_HUMAN	L	E	Y	L	T	A	N	I	L	E	K	V	G	K	E	V	K	N	S	C	R	L	C	I	T	P	E	H	V	K	R	A	L	Q	K	D	E	Q	L	R	W	I	L	E	L	E	D	D	T	H	S	Q	V	E	E	M	P	Q	S	E	114
547	Q71UI9	H2AV_HUMAN	L	E	Y	L	T	A	E	V	L	E	L	A	G	N	A	S	K	D	L	K	V	K	R	I	T	P	R	H	L	Q	L	A	I	R	G	D	E	E	L	D	S	L	I	-	K	A	T	I	A	G	G	G	V	I	P	H	I	H	K	S	117
559	P0C0S5	H2AZ_HUMAN	L	E	Y	L	T	A	E	V	L	E	L	A	G	N	A	S	K	D	L	K	V	K	R	I	T	P	R	H	L	Q	L	A	I	R	G	D	E	E	L	D	S	L	I	-	K	A	T	I	A	G	G	G	V	I	P	H	I	H	K	S	117

Info Page | Histone Modification List | Human Histone List | #H1 | #H2A | #H2B | #H3 | #H4 | Entries Featured in Article

Histone family

Example Page at Protein Sequence Level

Worksheets 4-8: Curated modification entries in human histone families (Part 3)

The next page demonstrates an example page of CHHM providing information of the modification entries at modification level.

To visualize the modification entries for each amino acid residue, clicking the “+” button on the left of the consecutive line, below the amino acid sequence of a histone variant will give the information of individual modification entries, including type, localization, source, and confidence level. For each modification entry, its type information is indicated at the beginning of each line of entries with the corresponding abbreviation. The italic letters/numbers indicate the sources of the entries, *i.e.*, knowledgebase/database or article. The combination of font color and background color denotes the highest confidence level of the retrieved records for each entry (refer to the key table for details).

Of note, the modification entries highlighted with a grey background color are only used to describe the situation in which the type of modification is identified, but the subtype of the modification has not been clarified. For instance, “sym/asym me2” is only used to describe the situation in which the dimethylation on this site is identified, but the type of methylation has not been clarified in the source knowledge/database.

This part of information is consistent to Figure 3 in manuscript.

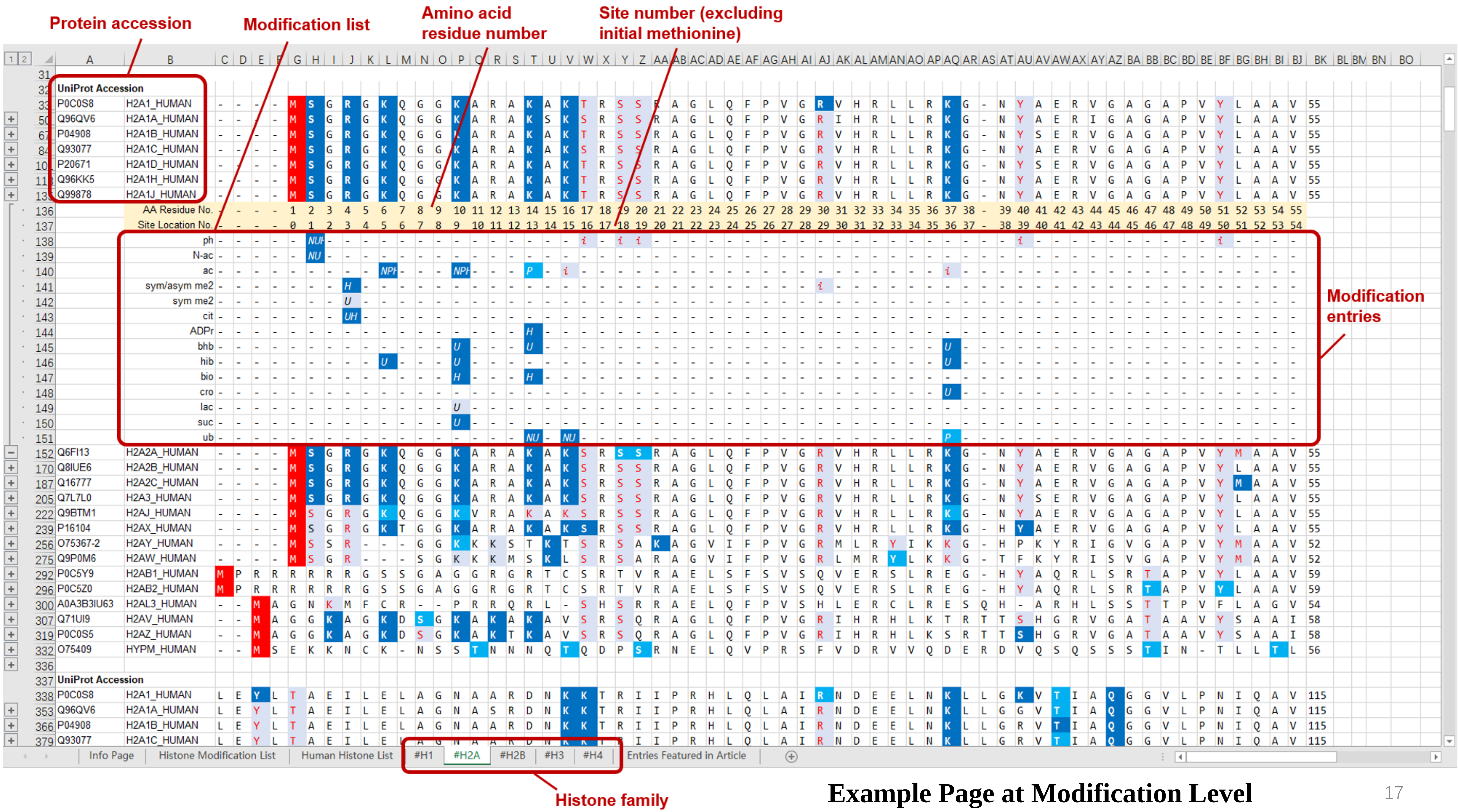

Example Page at Modification Level

Worksheet 9: List of modification entries featured in three articles

This worksheets provides the detailed information retrieved from articles, including source, confidence level, protein information, site information and modification/crosslink type. This part of information is included in the principal content of CHHM in the worksheets 4-8.

	A	B	C	D	E	F	G	H	I	J
1	**No.**	**Reference Article**	**Link**	**Experimental Evidence**	**UniProt Primary Accession**	**UniProt Accession Suffix**	**Protein Name**	**Site**	**Modification/Crosslink**	**Note**
2	1	Stützer et al., *Nat. Commun.* , 2020	https://www.ncbi.nlm.nih.gov/pmc/article	HTP	P10412	H14_HUMAN	Histone H1.4	K33	crosslink_C	ACGT-NH3
3		(UV-induced histone-DNA crosslinks			P10412	H14_HUMAN	Histone H1.4	P37/P38	crosslink_T	AATT
4		in H1 family)			P10412	H14_HUMAN	Histone H1.4	P37	crosslink_T	T-H3PO4
5					P10412	H14_HUMAN	Histone H1.4	K45	crosslink_C	CC-NH3
6					P10412	H14_HUMAN	Histone H1.4	K62	crosslink_C	CT-NH3
7					P10412	H14_HUMAN	Histone H1.4	K63	crosslink_C	CG-NH3
8					P10412	H14_HUMAN	Histone H1.4	K74	crosslink_C	CT-NH3
9					P10412	H14_HUMAN	Histone H1.4	K80	crosslink_C	ACG-NH3
10					P10412	H14_HUMAN	Histone H1.4	K84	crosslink_C	ACGT-NH3
11					P10412	H14_HUMAN	Histone H1.4	K89	crosslink_C	CG-NH3
12					P10412	H14_HUMAN	Histone H1.4	K105	crosslink_T	T-H3PO4
13					P10412	H14_HUMAN	Histone H1.4	K105	crosslink_C	ACCG-NH3
14					P10412	H14_HUMAN	Histone H1.4	K109	crosslink_C	ACCG-NH3
15					P10412	H14_HUMAN	Histone H1.4	K128	crosslink_C	ACCG-NH3
16					P10412	H14_HUMAN	Histone H1.4	K135	crosslink_C	ACC-NH3
17					P10412	H14_HUMAN	Histone H1.4	K139	crosslink_C	ACCG-NH3
18					P10412	H14_HUMAN	Histone H1.4	K147	crosslink_C	ACCT-NH3
19					P10412	H14_HUMAN	Histone H1.4	K156	crosslink_C	ACT-NH3
20					P10412	H14_HUMAN	Histone H1.4	K167	crosslink_C	ACCG-NH3
21					P10412	H14_HUMAN	Histone H1.4	K174/K176	crosslink_C	ACCG-NH3
22					P10412	H14_HUMAN	Histone H1.4	K191	crosslink_C	ACT-NH3
[illegible]					[illegible]	[illegible]	[illegible]	[illegible]	[illegible]	[illegible]

Info Page | Histone Modification List | Human Histone List | #H1 | #H2A | #H2B | #H3 | #H4 | Entries Featured in Article

3. How to use CHHM

HOW TO USE CHHM

To demonstrate how to use this tool, we provide the procedures in two assumptive sceneries:

Scenery A: investigation of the identified modifications on a target site on a histone variant.

For example, through site discovery, we have identified that **the site K5 on histone variant Q99878|H2A1J_HUMAN** is a site for a novel modification X, now we need to search for the identified modifications on the same site to investigate the potential influence of X on bioprocess.

Scenery B: investigation of the identified sites for a target modification on histone variants.

For example, we have discovered that **2-hydroxyisobutyrylation (hib) on H2A histone variants** contributes to some novel functions in chromatin architecture and thus has influence in gene expression, now we need to search for the identified sites for hib on H2A histone family to investigate how the mechanism works.

SCENERY A:

Investigation of the identified modifications on the site K5 on histone variant Q99878|H2A1J_HUMAN.

Step 1. Locate the target histone variant in the corresponding family "#H1/H2A/H2B/H3/H4" (worksheet).

Step 2. Find the target site on the sequence of histone variant and view information at protein sequence level.

Step 3. Click on the "+" button on the left of the consecutive line below the amino acid sequence of a histone variant to access the information of modification entries.

Step 4. Find the information of modification type and confidence level on the target site by viewing the modification entries.

Step 5. Check the annotations of knowledgebases/databases or articles denoting the sources of information, search for the original page on the corresponding knowledgebases/databases or articles for more details.

Step 1. Locate the target histone variant in the corresponding family "#H1/H2A/H2B/H3/H4" (worksheet).

	A	B	C	D	E	F	G	H	I	J	K	L	M	N	O	P	Q	R	S	T	U	V	W	X	Y	Z	AA	AB	AC	AD	AE	AF	AG	AH	AI	AJ	AK	AL	AM	AN	AO	AP	AQ	AR	AS	AT
31																																														
32	**UniProt Accession**																																													
33	P0C0S8	H2A1_HUMAN	-	-	-	-	M	S	G	R	G	K	Q	G	G	K	A	R	A	K	A	K	T	R	S	S	R	A	G	L	Q	F	P	V	G	R	V	H	R	L	L	R	K	G	-	N
50	Q96QV6	H2A1A_HUMAN	-	-	-	-	M	S	G	R	G	K	Q	G	G	K	A	R	A	K	S	K	S	R	S	S	R	A	G	L	Q	F	P	V	G	R	I	H	R	L	L	R	K	G	-	N
67	P04908	H2A1B_HUMAN	-	-	-	-	M	S	G	R	G	K	Q	G	G	K	A	R	A	K	A	K	T	R	S	S	R	A	G	L	Q	F	P	V	G	R	V	H	R	L	L	R	K	G	-	N
84	Q93077	H2A1C_HUMAN	-	-	-	-	M	S	G	R	G	K	Q	G	G	K	A	R	A	K	A	K	S	R	S	S	R	A	G	L	Q	F	P	V	G	R	V	H	R	L	L	R	K	G	-	N
101	P20671	H2A1D_HUMAN	-	-	-	-	M	S	G	R	G	K	Q	G	G	K	A	R	A	K	A	K	T	R	S	S	R	A	G	L	Q	F	P	V	G	R	V	H	R	L	L	R	K	G	-	N
118	Q96KK5	H2A1H_HUMAN	-	-	-	-	M	S	G	R	G	K	Q	G	G	K	A	R	A	K	A	K	T	R	S	S	R	A	G	L	Q	F	P	V	G	R	V	H	R	L	L	R	K	G	-	N
135	Q99878	H2A1J_HUMAN	-	-	-	-	M	S	G	R	G	K	Q	G	G	K	A	R	A	K	A	K	T	R	S	S	R	A	G	L	Q	F	P	V	G	R	V	H	R	L	L	R	K	G	-	N
152	Q6FI13	H2A2A_HUMAN	-	-	-	-	M	S	G	R	G	K	Q	G	G	K	A	R	A	K	A	K	S	R	S	S	R	A	G	L	Q	F	P	V	G	R	V	H	R	L	L	R	K	G	-	N
170	Q8IUE6	H2A2B_HUMAN	-	-	-	-	M	S	G	R	G	K	Q	G	G	K	A	R	A	K	A	K	S	R	S	S	R	A	G	L	Q	F	P	V	G	R	V	H	R	L	L	R	K	G	-	N
187	Q16777	H2A2C_HUMAN	-	-	-	-	M	S	G	R	G	K	Q	G	G	K	A	R	A	K	A	K	S	R	S	S	R	A	G	L	Q	F	P	V	G	R	V	H	R	L	L	R	K	G	-	N
205	Q7L7L0	H2A3_HUMAN	-	-	-	-	M	S	G	R	G	K	Q	G	G	K	A	R	A	K	A	K	S	R	S	S	R	A	G	L	Q	F	P	V	G	R	V	H	R	L	L	R	K	G	-	N
222	Q9BTM1	H2AJ_HUMAN	-	-	-	-	M	S	G	R	G	K	Q	G	G	K	V	R	A	K	A	K	S	R	S	S	R	A	G	L	Q	F	P	V	G	R	V	H	R	L	L	R	K	G	-	N
239	P16104	H2AX_HUMAN	-	-	-	-	M	S	G	R	G	K	T	G	G	K	A	R	A	K	A	K	S	R	S	S	R	A	G	L	Q	F	P	V	G	R	V	H	R	L	L	R	K	G	-	H
256	O75367-2	H2AY_HUMAN	-	-	-	-	M	S	S	R	-	-	-	G	G	K	K	K	S	T	K	T	S	R	S	A	K	A	G	V	I	F	P	V	G	R	M	L	R	Y	I	K	K	G	-	H
275	Q9P0M6	H2AW_HUMAN	-	-	-	-	M	S	G	R	-	-	-	S	G	K	K	K	M	S	K	L	S	R	S	A	R	A	G	V	I	F	P	V	G	R	L	M	R	Y	L	K	K	G	-	T
292	P0C5Y9	H2AB1_HUMAN	M	P	R	R	R	R	R	R	G	S	S	G	A	G	G	R	G	R	T	C	S	R	T	V	R	A	E	L	S	F	S	V	S	Q	V	E	R	S	L	R	E	G	-	H
296	P0C5Z0	H2AB2_HUMAN	M	P	R	R	R	R	R	R	G	S	S	G	A	G	G	R	G	R	T	C	S	R	T	V	R	A	E	L	S	F	S	V	S	Q	V	E	R	S	L	R	E	G	-	H
300	A0A3B3IU63	H2AL3_HUMAN	-	-	M	A	G	N	K	M	F	C	R	-	-	P	R	R	Q	R	L	-	S	H	S	R	R	A	E	L	Q	F	P	V	S	H	L	E	R	C	L	R	E	S	Q	H
307	Q71UI9	H2AV_HUMAN	-	-	M	A	G	G	K	A	G	K	D	S	G	K	A	K	A	K	A	V	S	R	S	Q	R	A	G	L	Q	F	P	V	G	R	I	H	R	H	L	K	T	R	T	T
319	P0C0S5	H2AZ_HUMAN	-	-	M	A	G	G	K	A	G	K	D	S	G	K	A	K	T	K	A	V	S	R	S	Q	R	A	G	L	Q	F	P	V	G	R	I	H	R	H	L	K	S	R	T	T
332	O75409	HYPM_HUMAN	-	-	M	S	E	K	K	N	C	K	-	N	S	S	T	N	N	N	Q	T	Q	D	P	S	R	N	E	L	Q	V	P	R	S	F	V	D	R	V	V	Q	D	E	R	D
336																																														
337	**UniProt Accession**																																													
338	P0C0S8	H2A1_HUMAN	L	E	Y	L	T	A	E	I	L	E	L	A	G	N	A	A	R	D	N	K	K	T	R	I	I	P	R	H	L	Q	L	A	I	R	N	D	E	E	L	N	K	L	L	G
353	Q96QV6	H2A1A_HUMAN	L	E	Y	L	T	A	E	I	L	E	L	A	G	N	A	S	R	D	N	K	K	T	R	I	I	P	R	H	L	Q	L	A	I	R	N	D	E	E	L	N	K	L	L	G
366	P04908	H2A1B_HUMAN	L	E	Y	L	T	A	E	I	L	E	L	A	G	N	A	A	R	D	N	K	K	T	R	I	I	P	R	H	L	Q	L	A	I	R	N	D	E	E	L	N	K	L	L	G
379	Q93077	H2A1C_HUMAN	L	E	Y	L	T	A	E	I	L	E	L	A	G	N	A	A	R	D	N	K	K	T	R	I	I	P	R	H	L	Q	L	A	I	R	N	D	E	E	L	N	K	L	L	G
392	P20671	H2A1D_HUMAN	L	E	Y	L	T	A	E	I	L	E	L	A	G	N	A	A	R	D	N	K	K	T	R	I	I	P	R	H	L	Q	L	A	I	R	N	D	E	E	L	N	K	L	L	G
407	Q96KK5	H2A1H_HUMAN	L	E	Y	L	T	A	E	I	L	E	L	A	G	N	A	A	R	D	N	K	K	T	R	I	I	P	R	H	L	Q	L	A	I	R	N	D	E	E	L	N	K	L	L	G
422	Q99878	H2A1J_HUMAN	L	E	Y	L	T	A	E	I	L	E	L	A	G	N	A	A	R	D	N	K	K	T	R	I	I	P	R	H	L	Q	L	A	I	R	N	D	E	E	L	N	K	L	L	G
437	Q6FI13	H2A2A_HUMAN	L	E	Y	L	T	A	E	I	L	E	L	A	G	N	A	A	R	D	N	K	K	T	R	I	I	P	R	H	L	Q	L	A	I	R	N	D	E	E	L	N	K	L	L	G
452	Q8IUE6	H2A2B_HUMAN	L	E	Y	L	T	A	E	I	L	E	L	A	G	N	A	A	R	D	N	K	K	T	R	I	I	P	R	H	L	Q	L	A	V	R	N	D	E	E	L	N	K	L	L	G
465	Q16777	H2A2C_HUMAN	L	E	Y	L	T	A	E	I	L	E	L	A	G	N	A	A	R	D	N	K	K	T	R	I	I	P	R	H	L	Q	L	A	I	R	N	D	E	E	L	N	K	L	L	G
480	Q7L7L0	H2A3_HUMAN	L	E	Y	L	T	A	E	I	L	E	L	A	G	N	A	A	R	D	N	K	K	T	R	I	I	P	R	H	L	Q	L	A	I	R	N	D	E	E	L	N	K	L	L	G
493	Q9BTM1	H2AJ_HUMAN	L	E	Y	L	T	A	E	I	L	E	L	A	G	N	A	A	R	D	N	K	K	T	R	I	I	P	R	H	L	Q	L	A	I	R	N	D	E	E	L	N	K	L	L	G
508	P16104	H2AX_HUMAN	L	E	Y	L	T	A	E	I	L	E	L	A	G	N	A	A	R	D	N	K	K	T	R	I	I	P	R	H	L	Q	L	A	I	R	N	D	E	E	L	N	K	L	L	G
521	O75367-2	H2AY_HUMAN	L	E	Y	L	T	A	E	I	L	E	L	A	G	N	A	A	R	D	N	K	K	G	R	V	T	P	R	H	I	L	L	A	V	A	N	D	E	E	L	N	Q	L	L	K
528	Q9P0M6	H2AW_HUMAN	I	E	Y	L	A	A	E	I	L	E	L	A	G	N	A	A	R	D	N	K	K	A	R	I	A	P	R	H	I	L	L	A	V	A	N	D	E	E	L	N	Q	L	L	K
535	P0C5Y9	H2AB1_HUMAN	I	E	Y	L	T	A	K	V	P	E	L	A	G	N	E	A	Q	N	S	G	E	R	N	I	T	P	L	L	L	D	M	V	V	H	N	D	R	L	L	S	T	L	F	N
539	P0C5Z0	H2AB2_HUMAN	I	E	Y	L	T	A	K	V	L	E	L	A	G	N	E	A	Q	N	S	G	E	R	N	I	T	P	L	L	L	D	M	V	V	H	N	D	R	L	L	S	T	L	F	N
543	A0A3B3IU63	H2AL3_HUMAN	L	E	Y	L	T	A	N	I	L	E	K	V	G	K	E	V	K	N	S	C	R	L	C	I	T	P	E	H	V	K	R	A	L	Q	K	D	E	Q	L	R	W	I	L	E
547	Q71UI9	H2AV_HUMAN	L	E	Y	L	T	A	E	V	L	E	L	A	G	N	A	S	K	D	L	K	V	K	R	I	T	P	R	H	L	Q	L	A	I	R	G	D	E	E	L	D	S	L	I	-
559	P0C0S5	H2AZ_HUMAN	L	E	Y	L	T	A	E	V	L	E	L	A	G	N	A	S	K	D	L	K	V	K	R	I	T	P	R	H	L	Q	L	A	I	R	G	D	E	E	L	D	S	L	I	-

Info Page | Histone Modification List | Human Histone List | #H1 | #H2A | #H2B | #H3 | #H4 | Entries Featured in Article

Step 2. Find the target site on the sequence of histone variant and view information at protein sequence level.

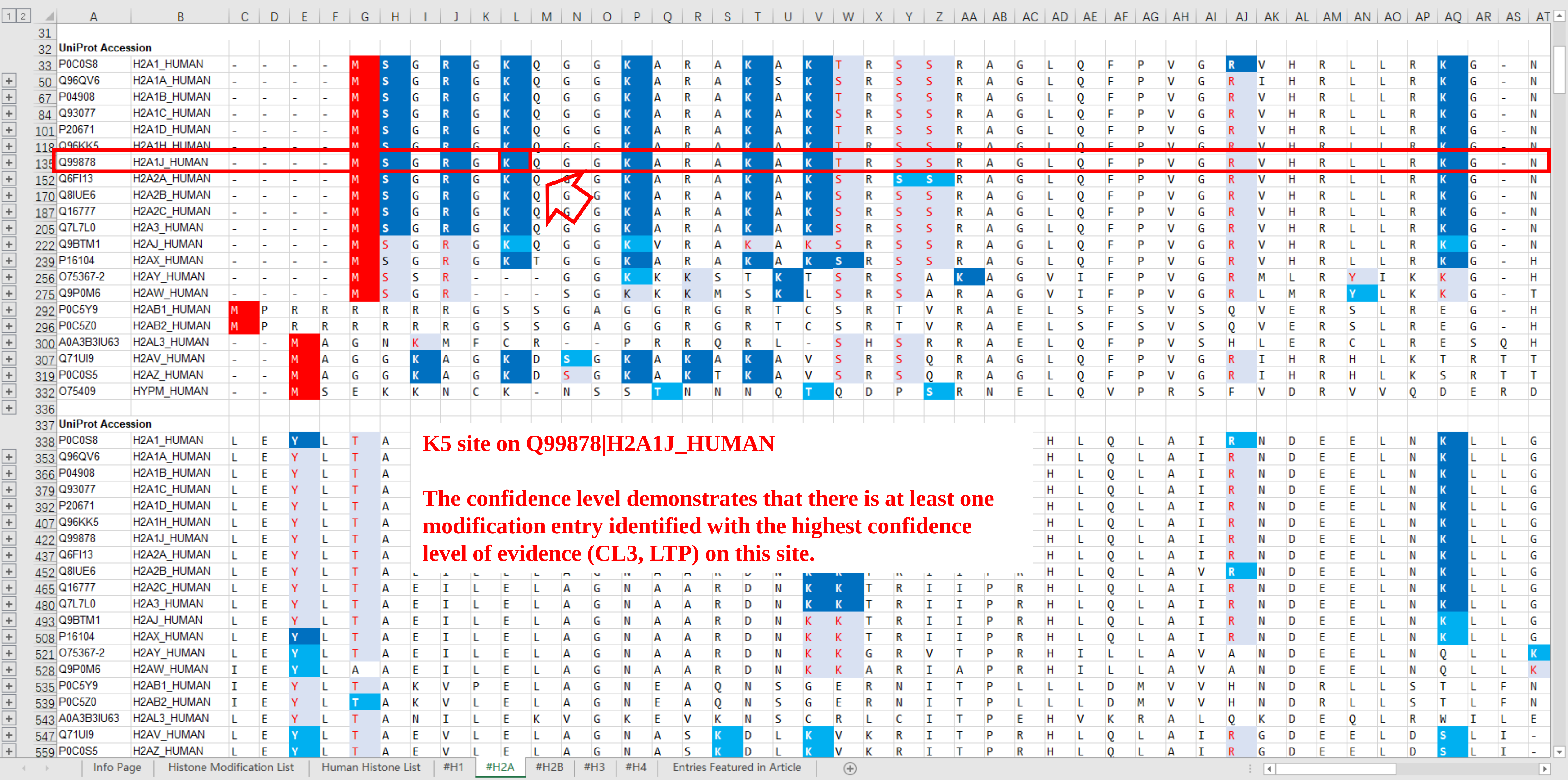

K5 site on Q99878|H2A1J_HUMAN

The confidence level demonstrates that there is at least one modification entry identified with the highest confidence level of evidence (CL3, LTP) on this site.

Step 3. Click on the "+" button on the left of the consecutive line below the amino acid sequence of a histone variant access the information of individual modification entries.

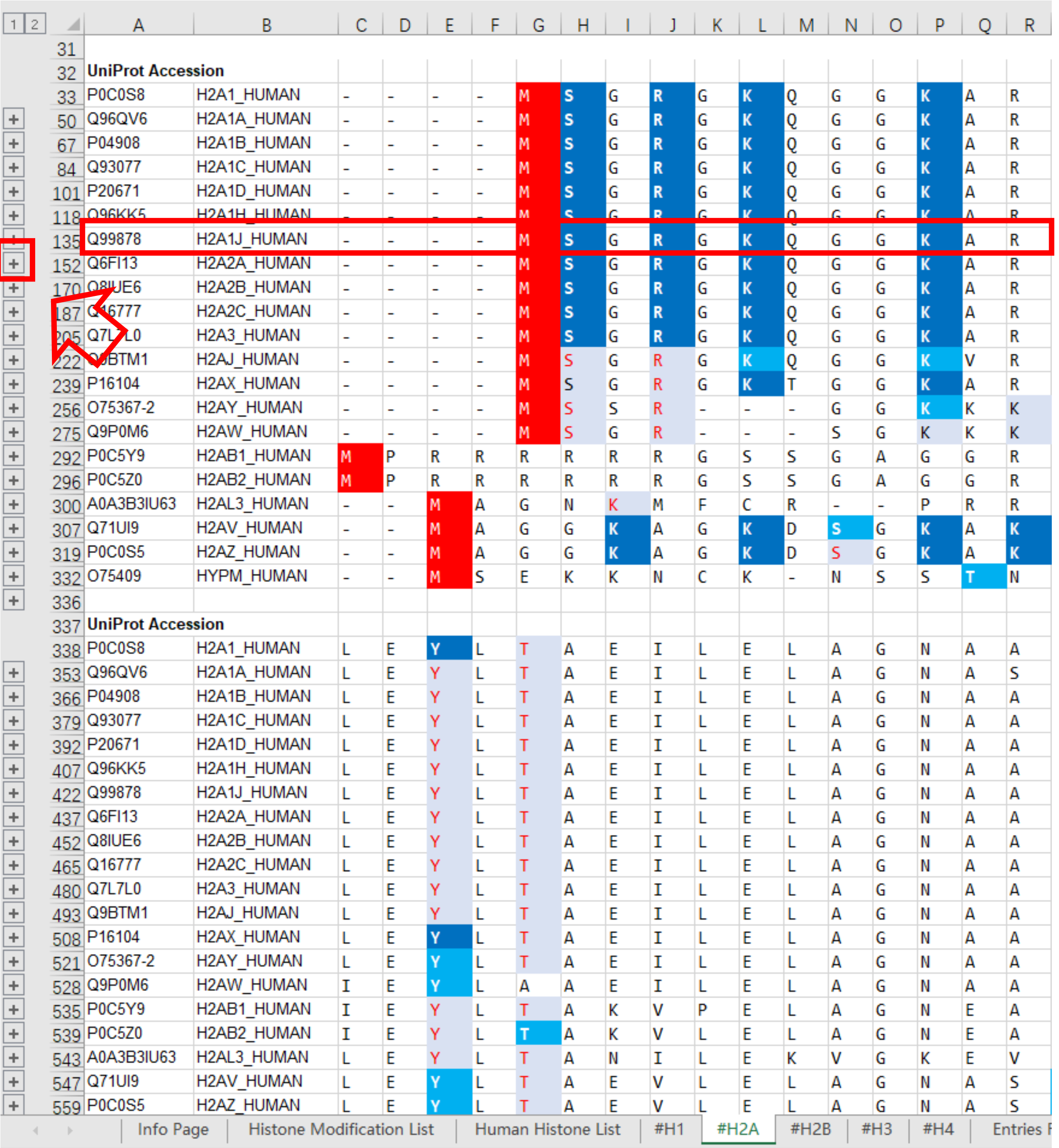

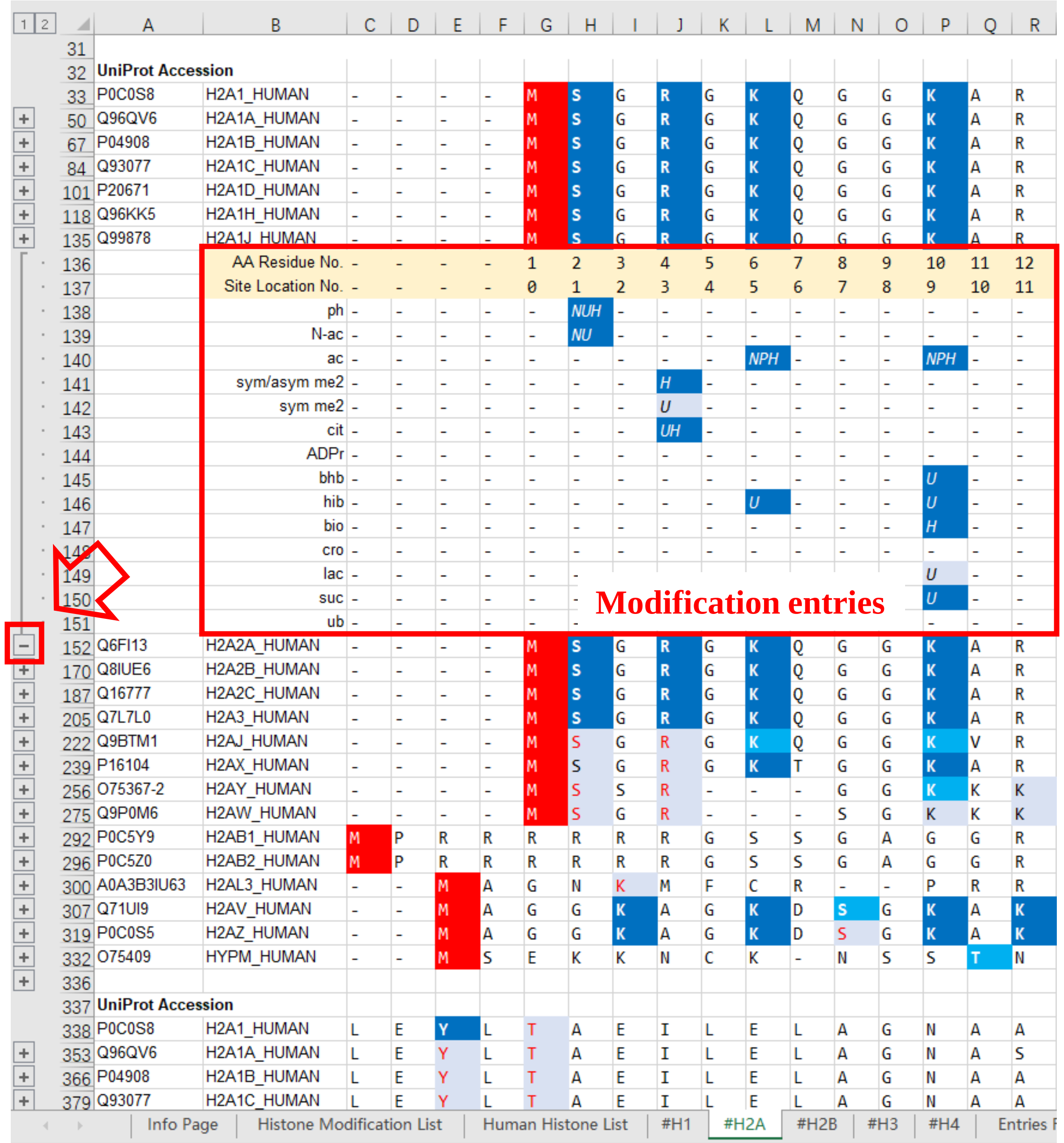

Step 4. Find the information of modification type and confidence level on the target site by viewing the modification entries.

	A	B	C	D	E	F	G	H	I	J	K	L	M	N	O	P	Q	R	S	T	U	V	W	X	Y	Z	AA	AB	AC	AD	AE	AF	AG	AH	AI	AJ	AK	AL	AM	AN	AO	AP	AQ	AR	AS	AT
31																																														
32	UniProt Accession																																													
33	P0C0S8	H2A1_HUMAN	-	-	-	-	M	S	G	R	G	K	Q	G	G	K	A	R	A	K	A	K	T	R	S	S	R	A	G	L	Q	F	P	V	G	R	V	H	R	L	L	R	K	G	-	N
50	Q96QV6	H2A1A_HUMAN	-	-	-	-	M	S	G	R	G	K	Q	G	G	K	A	R	A	K	S	K	S	R	S	S	R	A	G	L	Q	F	P	V	G	R	I	H	R	L	L	R	K	G	-	N
67	P04908	H2A1B_HUMAN	-	-	-	-	M	S	G	R	G	K	Q	G	G	K	A	R	A	K	A	K	T	R	S	S	R	A	G	L	Q	F	P	V	G	R	V	H	R	L	L	R	K	G	-	N
84	Q93077	H2A1C_HUMAN	-	-	-	-	M	S	G	R	G	K	Q	G	G	K	A	R	A	K	A	K	S	R	S	S	R	A	G	L	Q	F	P	V	G	R	V	H	R	L	L	R	K	G	-	N
101	P20671	H2A1D_HUMAN	-	-	-	-	M	S	G	R	G	K	Q	G	G	K	A	R	A	K	A	K	T	R	S	S	R	A	G	L	Q	F	P	V	G	R	V	H	R	L	L	R	K	G	-	N
118	Q96KK5	H2A1H_HUMAN	-	-	-	-	M	S	G	R	G	K	Q	G	G	K	A	R	A	K	A	K	T	R	S	S	R	A	G	L	Q	F	P	V	G	R	V	H	R	L	L	R	K	G	-	N
135	Q99878	H2A1J_HUMAN	-	-	-	-	M	S	G	R	G	K	Q	G	G	K	A	R	A	K	A	K	T	R	S	S	R	A	G	L	Q	F	P	V	G	R	V	H	R	L	L	R	K	G	-	N
136		AA Residue No.	-	-	-	-	1	2	3	4	5	6	7	8	9	10	11	12	13	14	15	16	17	18	19	20	21	22	23	24	25	26	27	28	29	30	31	32	33	34	35	36	37	38	-	39
137		Site Location No.	-	-	-	-	0	1	2	3	4	5	6	7	8	9	10	11	12	13	14	15	16	17	18	19	20	21	22	23	24	25	26	27	28	29	30	31	32	33	34	35	36	37	-	38
138		ph	-	-	-	-	-	NUH	-	-	-	-	-	-	-	-	-	-	-	-	-	-	i	-	i	i	-	-	-	-	-	-	-	-	-	-	-	-	-	-	-	-	-	-	-	-
139		N-ac	-	-	-	-	-	NU	-	-	-	-	-	-	-	-	-	-	-	-	-	-	-	-	-	-	-	-	-	-	-	-	-	-	-	-	-	-	-	-	-	-	-	-	-	-
140		ac	-	-	-	-	-	-	-	-	-	NPH	-	-	-	NPH	-	-	-	P	-	i	-	-	-	-	-	-	-	-	-	-	-	-	-	-	-	-	-	-	-	-	i	-	-	-
141		sym/asym me2	-	-	-	-	-	-	-	H	-	-	-	-	-	-	-	-	-	-	-	-	-	-	-	-	-	-	-	-	-	-	-	-	-	i	-	-	-	-	-	-	-	-	-	-
142		sym me2	-	-	-	-	-	-	-	U	-	-	-	-	-	-	-	-	-	-	-	-	-	-	-	-	-	-	-	-	-	-	-	-	-	-	-	-	-	-	-	-	-	-	-	-
143		cit	-	-	-	-	-	-	-	UH	-	-	-	-	-	-	-	-	-	-	-	-	-	-	-	-	-	-	-	-	-	-	-	-	-	-	-	-	-	-	-	-	-	-	-	-
144		ADPr	-	-	-	-	-	-	-	-	-	-	-	-	[illegible]	[illegible]	[illegible]	[illegible]	[illegible]	[illegible]	[illegible]	[illegible]	[illegible]	[illegible]	[illegible]	[illegible]	[illegible]	[illegible]	[illegible]	[illegible]	[illegible]	[illegible]	[illegible]	[illegible]	[illegible]	[illegible]	[illegible]	[illegible]	[illegible]	[illegible]	-	-	-	-	-	-
145		bhb	-	-	-	-	-	-	-	-	-	-	-	-	[illegible]	[illegible]	[illegible]	[illegible]	[illegible]	[illegible]	[illegible]	[illegible]	[illegible]	[illegible]	[illegible]	[illegible]	[illegible]	[illegible]	[illegible]	[illegible]	[illegible]	[illegible]	[illegible]	[illegible]	[illegible]	[illegible]	[illegible]	[illegible]	[illegible]	[illegible]	-	-	U	-	-	-
146		hib	-	-	-	-	-	-	-	-	-	U	-	-	[illegible]	[illegible]	[illegible]	[illegible]	[illegible]	[illegible]	[illegible]	[illegible]	[illegible]	[illegible]	[illegible]	[illegible]	[illegible]	[illegible]	[illegible]	[illegible]	[illegible]	[illegible]	[illegible]	[illegible]	[illegible]	[illegible]	[illegible]	[illegible]	[illegible]	[illegible]	-	-	U	-	-	-
147		bio	-	-	-	-	-	-	-	-	-	-	-	-	[illegible]	[illegible]	[illegible]	[illegible]	[illegible]	[illegible]	[illegible]	[illegible]	[illegible]	[illegible]	[illegible]	[illegible]	[illegible]	[illegible]	[illegible]	[illegible]	[illegible]	[illegible]	[illegible]	[illegible]	[illegible]	[illegible]	[illegible]	[illegible]	[illegible]	[illegible]	-	-	-	-	-	-
148		cro	-	-	-	-	-	-	-	-	-	-	-	-	[illegible]	[illegible]	[illegible]	[illegible]	[illegible]	[illegible]	[illegible]	[illegible]	[illegible]	[illegible]	[illegible]	[illegible]	[illegible]	[illegible]	[illegible]	[illegible]	[illegible]	[illegible]	[illegible]	[illegible]	[illegible]	[illegible]	[illegible]	[illegible]	[illegible]	[illegible]	-	-	U	-	-	-
149		lac	-	-	-	-	-	-	-	-	-	-	-	-	[illegible]	[illegible]	[illegible]	[illegible]	[illegible]	[illegible]	[illegible]	[illegible]	[illegible]	[illegible]	[illegible]	[illegible]	[illegible]	[illegible]	[illegible]	[illegible]	[illegible]	[illegible]	[illegible]	[illegible]	[illegible]	[illegible]	[illegible]	[illegible]	[illegible]	[illegible]	-	-	-	-	-	-
150		suc	-	-	-	-	-	-	-	-	-	-	-	-	-	U	-	-	-	-	-	-	-	-	-	-	-	-	-	-	-	-	-	-	-	-	-	-	-	-	-	-	-	-	-	-
151		ub	-	-	-	-	-	-	-	-	-	-	-	-	-	-	-	-	-	NU	-	NU	-	-	-	-	-	-	-	-	-	-	-	-	-	-	-	-	-	-	-	-	P	-	-	-
152	Q6FI13	H2A2A_HUMAN	-	-	-	-	M	S	G	R	G	K	Q	G	G	K	A	R	A	K	A	K	S	R	S	S	R	A	G	L	Q	F	P	V	G	R	V	H	R	L	L	R	K	G	-	N
170	Q8IUE6	H2A2B_HUMAN	-	-	-	-	M	S	G	R	G	K	Q	G	G	K	A	R	A	K	A	K	S	R	S	S	R	A	G	L	Q	F	P	V	G	R	V	H	R	L	L	R	K	G	-	N
187	Q16777	H2A2C_HUMAN	-	-	-	-	M	S	G	R	G	K	Q	G	G	K	A	R	A	K	A	K	S	R	S	S	R	A	G	L	Q	F	P	V	G	R	V	H	R	L	L	R	K	G	-	N
205	Q7L7L0	H2A3_HUMAN	-	-	-	-	M	S	G	R	G	K	Q	G	G	K	A	R	A	K	A	K	S	R	S	S	R	A	G	L	Q	F	P	V	G	R	V	H	R	L	L	R	K	G	-	N
222	Q9BTM1	H2AJ_HUMAN	-	-	-	-	M	S	G	R	G	K	Q	G	G	K	V	R	A	K	A	K	S	R	S	S	R	A	G	L	Q	F	P	V	G	R	V	H	R	L	L	R	K	G	-	N
239	P16104	H2AX_HUMAN	-	-	-	-	M	S	G	R	G	K	T	G	G	K	A	R	A	K	A	K	S	R	S	S	R	A	G	L	Q	F	P	V	G	R	V	H	R	L	L	R	K	G	-	H
256	O75367-2	H2AY_HUMAN	-	-	-	-	M	S	S	R	-	-	-	G	G	K	K	K	S	T	K	T	S	R	S	A	K	A	G	V	I	F	P	V	G	R	M	L	R	Y	I	K	K	G	-	H
275	Q9P0M6	H2AW_HUMAN	-	-	-	-	M	S	G	R	-	-	-	S	G	K	K	K	M	S	K	L	S	R	S	A	R	A	G	V	I	F	P	V	G	R	L	M	R	Y	L	K	K	G	-	T
292	P0C5Y9	H2AB1_HUMAN	M	P	R	R	R	R	R	R	G	S	S	G	A	G	G	R	G	R	T	C	S	R	T	V	R	A	E	L	S	F	S	V	S	Q	V	E	R	S	L	R	E	G	-	H
296	P0C5Z0	H2AB2_HUMAN	M	P	R	R	R	R	R	R	G	S	S	G	A	G	G	R	G	R	T	C	S	R	T	V	R	A	E	L	S	F	S	V	S	Q	V	E	R	S	L	R	E	G	-	H
300	A0A3B3IU63	H2AL3_HUMAN	-	-	M	A	G	N	K	M	F	C	R	-	-	P	R	R	Q	R	L	-	S	H	S	R	R	A	E	L	Q	F	P	V	S	H	L	E	R	C	L	R	E	S	Q	H
307	Q71UI9	H2AV_HUMAN	-	-	M	A	G	G	K	A	G	K	D	S	G	K	A	K	A	K	A	V	S	R	S	Q	R	A	G	L	Q	F	P	V	G	R	I	H	R	H	L	K	T	R	T	T
319	P0C0S5	H2AZ_HUMAN	-	-	M	A	G	G	K	A	G	K	D	S	G	K	A	K	T	K	A	V	S	R	S	Q	R	A	G	L	Q	F	P	V	G	R	I	H	R	H	L	K	S	R	T	T
332	O75409	HYPM_HUMAN	-	-	M	S	E	K	K	N	C	K	-	N	S	S	T	N	N	N	Q	T	Q	D	P	S	R	N	E	L	Q	V	P	R	S	F	V	D	R	V	V	Q	D	E	R	D
336																																														
337	UniProt Accession																																													
338	P0C0S8	H2A1_HUMAN	L	E	Y	L	T	A	E	I	L	E	L	A	G	N	A	A	R	D	N	K	K	T	R	I	I	P	R	H	L	Q	L	A	I	R	N	D	E	E	L	N	K	L	L	G
353	Q96QV6	H2A1A_HUMAN	L	E	Y	L	T	A	E	I	L	E	L	A	G	N	A	S	R	D	N	K	K	T	R	I	I	P	R	H	L	Q	L	A	I	R	N	D	E	E	L	N	K	L	L	G
366	P04908	H2A1B_HUMAN	L	E	Y	L	T	A	E	I	L	E	L	A	G	N	A	A	R	D	N	K	K	T	R	I	I	P	R	H	L	Q	L	A	I	R	N	D	E	E	L	N	K	L	L	G
379	Q93077	H2A1C_HUMAN	L	E	Y	L	T	A	E	I	L	E	L	A	G	N	A	A	R	D	N	K	K	T	R	I	I	P	R	H	L	Q	L	A	I	R	N	D	E	E	L	N	K	L	L	G

Info Page | Histone Modification List | Human Histone List | #H1 | #H2A | #H2B | #H3 | #H4 | Entries Featured in Article

It is found that K5 on histone variant Q99878|H2A1J_HUMAN is identified as a site for N^{ε}-acetylation (ac) and 2-hydroxyisobutyrylation (hib) with the highest confidence level of evidence (CL3, LTP).

Step 5. Check the annotations of knowledgebases/databases or articles denoting the sources of information, search for the original page on the corresponding knowledgebases/databases or articles for more details.

	A	B	C	D	E	F	G	H	I	J	K	L	M	N	O	P	Q	R	S	T	U	V	W	X	Y	Z	AA	AB	AC	AD	AE	AF	AG	AH	AI	AJ	AK	AL	AM	AN	AO	AP	AQ	AR	AS	AT
31																																														
32	UniProt Accession																																													
33	P0C0S8	H2A1_HUMAN	-	-	-	-	M	S	G	R	G	K	Q	G	G	K	A	R	A	K	A	K	T	R	S	S	R	A	G	L	Q	F	P	V	G	R	V	H	R	L	L	R	K	G	-	N
50	Q96QV6	H2A1A_HUMAN	-	-	-	-	M	S	G	R	G	K	Q	G	G	K	A	R	A	K	S	K	S	R	S	S	R	A	G	L	Q	F	P	V	G	R	I	H	R	L	L	R	K	G	-	N
67	P04908	H2A1B_HUMAN	-	-	-	-	M	S	G	R	G	K	Q	G	G	K	A	R	A	K	A	K	T	R	S	S	R	A	G	L	Q	F	P	V	G	R	V	H	R	L	L	R	K	G	-	N
84	Q93077	H2A1C_HUMAN	-	-	-	-	M	S	G	R	G	K	Q	G	G	K	A	R	A	K	A	K	S	R	S	S	R	A	G	L	Q	F	P	V	G	R	V	H	R	L	L	R	K	G	-	N
101	P20671	H2A1D_HUMAN	-	-	-	-	M	S	G	R	G	K	Q	G	G	K	A	R	A	K	A	K	T	R	S	S	R	A	G	L	Q	F	P	V	G	R	V	H	R	L	L	R	K	G	-	N
118	Q96KK5	H2A1H_HUMAN	-	-	-	-	M	S	G	R	G	K	Q	G	G	K	A	R	A	K	A	K	T	R	S	S	R	A	G	L	Q	F	P	V	G	R	V	H	R	L	L	R	K	G	-	N
135	Q99878	H2A1J_HUMAN	-	-	-	-	M	S	G	R	G	K	Q	G	G	K	A	R	A	K	A	K	T	R	S	S	R	A	G	L	Q	F	P	V	G	R	V	H	R	L	L	R	K	G	-	N
136		AA Residue No.	-	-	-	-	1	2	3	4	5	6	7	8	9	10	11	12	13	14	15	16	17	18	19	20	21	22	23	24	25	26	27	28	29	30	31	32	33	34	35	36	37	38	-	39
137		Site Location No.	-	-	-	-	0	1	2	3	4	5	6	7	8	9	10	11	12	13	14	15	16	17	18	19	20	21	22	23	24	25	26	27	28	29	30	31	32	33	34	35	36	37	-	38
138		ph	-	-	-	-	-	NUH	-	-	-	-	-	-	-	-	-	-	-	-	-	-	i	-	i	i	-	-	-	-	-	-	-	-	-	-	-	-	-	-	-	-	-	-	-	-
139		N-ac	-	-	-	-	-	NU	-	-	-	-	-	-	-	-	-	-	-	-	-	-	-	-	-	-	-	-	-	-	-	-	-	-	-	-	-	-	-	-	-	-	-	-	-	-
140		ac	-	-	-	-	-	-	-	-	-	NPH	-	-	[illegible]	[illegible]	[illegible]	[illegible]	[illegible]	[illegible]	[illegible]	[illegible]	[illegible]	[illegible]	[illegible]	[illegible]	[illegible]	[illegible]	[illegible]	[illegible]	[illegible]	[illegible]	[illegible]	[illegible]	[illegible]	[illegible]	[illegible]	[illegible]	[illegible]	[illegible]	[illegible]	[illegible]	i	-	-	-
141		sym/asym me2	-	-	-	-	-	-	-	H	-	-	-	-	[illegible]	[illegible]	[illegible]	[illegible]	[illegible]	[illegible]	[illegible]	[illegible]	[illegible]	[illegible]	[illegible]	[illegible]	[illegible]	[illegible]	[illegible]	[illegible]	[illegible]	[illegible]	[illegible]	[illegible]	[illegible]	[illegible]	[illegible]	[illegible]	[illegible]	[illegible]	[illegible]	[illegible]	-	-	-	-
142		sym me2	-	-	-	-	-	-	-	U	-	-	-	-	[illegible]	[illegible]	[illegible]	[illegible]	[illegible]	[illegible]	[illegible]	[illegible]	[illegible]	[illegible]	[illegible]	[illegible]	[illegible]	[illegible]	[illegible]	[illegible]	[illegible]	[illegible]	[illegible]	[illegible]	[illegible]	[illegible]	[illegible]	[illegible]	[illegible]	[illegible]	[illegible]	[illegible]	-	-	-	-
143		cit	-	-	-	-	-	-	-	UH	-	-	-	-	[illegible]	[illegible]	[illegible]	[illegible]	[illegible]	[illegible]	[illegible]	[illegible]	[illegible]	[illegible]	[illegible]	[illegible]	[illegible]	[illegible]	[illegible]	[illegible]	[illegible]	[illegible]	[illegible]	[illegible]	[illegible]	[illegible]	[illegible]	[illegible]	[illegible]	[illegible]	[illegible]	[illegible]	-	-	-	-
144		ADPr	-	-	-	-	-	-	-	-	-	-	-	-	[illegible]	[illegible]	[illegible]	[illegible]	[illegible]	[illegible]	[illegible]	[illegible]	[illegible]	[illegible]	[illegible]	[illegible]	[illegible]	[illegible]	[illegible]	[illegible]	[illegible]	[illegible]	[illegible]	[illegible]	[illegible]	[illegible]	[illegible]	[illegible]	[illegible]	[illegible]	[illegible]	[illegible]	-	-	-	-
145		bhb	-	-	-	-	-	-	-	-	-	-	-	-	[illegible]	[illegible]	[illegible]	[illegible]	[illegible]	[illegible]	[illegible]	[illegible]	[illegible]	[illegible]	[illegible]	[illegible]	[illegible]	[illegible]	[illegible]	[illegible]	[illegible]	[illegible]	[illegible]	[illegible]	[illegible]	[illegible]	[illegible]	[illegible]	[illegible]	[illegible]	[illegible]	[illegible]	U	-	-	-
146		hib	-	-	-	-	-	-	-	-	-	U	-	-	[illegible]	[illegible]	[illegible]	[illegible]	[illegible]	[illegible]	[illegible]	[illegible]	[illegible]	[illegible]	[illegible]	[illegible]	[illegible]	[illegible]	[illegible]	[illegible]	[illegible]	[illegible]	[illegible]	[illegible]	[illegible]	[illegible]	[illegible]	[illegible]	[illegible]	[illegible]	[illegible]	[illegible]	U	-	-	-
147		bio	-	-	-	-	-	-	-	-	-	-	-	-	-	H	-	-	-	H	-	-	-	-	-	-	-	-	-	-	-	-	-	-	-	-	-	-	-	-	-	-	-	-	-	-
148		cro	-	-	-	-	-	-	-	-	-	-	-	-	-	-	-	-	-	-	-	-	-	-	-	-	-	-	-	-	-	-	-	-	-	-	-	-	-	-	-	-	U	-	-	-
149		lac	-	-	-	-	-	-	-	-	-	-	-	-	-	U	-	-	-	-	-	-	-	-	-	-	-	-	-	-	-	-	-	-	-	-	-	-	-	-	-	-	-	-	-	-
150		suc	-	-	-	-	-	-	-	-	-	-	-	-	-	U	-	-	-	-	-	-	-	-	-	-	-	-	-	-	-	-	-	-	-	-	-	-	-	-	-	-	-	-	-	-
151		ub	-	-	-	-	-	-	-	-	-	-	-	-	-	-	-	-	-	NU	-	NU	-	-	-	-	-	-	-	-	-	-	-	-	-	-	-	-	-	-	-	-	P	-	-	-
152	Q6FI13	H2A2A_HUMAN	-	-	-	-	M	S	G	R	G	K	Q	G	G	K	A	R	A	K	A	K	S	R	S	S	R	A	G	L	Q	F	P	V	G	R	V	H	R	L	L	R	K	G	-	N
170	Q8IUE6	H2A2B_HUMAN	-	-	-	-	M	S	G	R	G	K	Q	G	G	K	A	R	A	K	A	K	S	R	S	S	R	A	G	L	Q	F	P	V	G	R	V	H	R	L	L	R	K	G	-	N
187	Q16777	H2A2C_HUMAN	-	-	-	-	M	S	G	R	G	K	Q	G	G	K	A	R	A	K	A	K	S	R	S	S	R	A	G	L	Q	F	P	V	G	R	V	H	R	L	L	R	K	G	-	N
205	Q7L7L0	H2A3_HUMAN	-	-	-	-	M	S	G	R	G	K	Q	G	G	K	A	R	A	K	A	K	S	R	S	S	R	A	G	L	Q	F	P	V	G	R	V	H	R	L	L	R	K	G	-	N
222	Q9BTM1	H2AJ_HUMAN	-	-	-	-	M	S	G	R	G	K	Q	G	G	K	V	R	A	K	A	K	S	R	S	S	R	A	G	L	Q	F	P	V	G	R	V	H	R	L	L	R	K	G	-	N
239	P16104	H2AX_HUMAN	-	-	-	-	M	S	G	R	G	K	T	G	G	K	A	R	A	K	A	K	S	R	S	S	R	A	G	L	Q	F	P	V	G	R	V	H	R	L	L	R	K	G	-	H
256	O75367-2	H2AY_HUMAN	-	-	-	-	M	S	S	R	-	-	-	G	G	K	K	K	S	T	K	T	S	R	S	A	K	A	G	V	I	F	P	V	G	R	M	L	R	Y	I	K	K	G	-	H
275	Q9P0M6	H2AW_HUMAN	-	-	-	-	M	S	G	R	-	-	-	S	G	K	K	K	M	S	K	L	S	R	S	A	R	A	G	V	I	F	P	V	G	R	L	M	R	Y	L	K	K	G	-	T
292	P0C5Y9	H2AB1_HUMAN	M	P	R	R	R	R	R	R	G	S	S	G	A	G	G	R	G	R	T	C	S	R	T	V	R	A	E	L	S	F	S	V	S	Q	V	E	R	S	L	R	E	G	-	H
296	P0C5Z0	H2AB2_HUMAN	M	P	R	R	R	R	R	R	G	S	S	G	A	G	G	R	G	R	T	C	S	R	T	V	R	A	E	L	S	F	S	V	S	Q	V	E	R	S	L	R	E	G	-	H
300	A0A3B3IU63	H2AL3_HUMAN	-	-	M	A	G	N	K	M	F	C	R	-	-	P	R	R	Q	R	L	-	S	H	S	R	R	A	E	L	Q	F	P	V	S	H	L	E	R	C	L	R	E	S	Q	H
307	Q71UI9	H2AV_HUMAN	-	-	M	A	G	G	K	A	G	K	D	S	G	K	A	K	A	K	A	V	S	R	S	Q	R	A	G	L	Q	F	P	V	G	R	I	H	R	H	L	K	T	R	T	T
319	P0C0S5	H2AZ_HUMAN	-	-	M	A	G	G	K	A	G	K	D	S	G	K	A	K	T	K	A	V	S	R	S	Q	R	A	G	L	Q	F	P	V	G	R	I	H	R	H	L	K	S	R	T	T
332	O75409	HYPM_HUMAN	-	-	M	S	E	K	K	N	C	K	-	N	S	S	T	N	N	N	Q	T	Q	D	P	S	R	N	E	L	Q	V	P	R	S	F	V	D	R	V	V	Q	D	E	R	D
336																																														
337	UniProt Accession																																													
338	P0C0S8	H2A1_HUMAN	L	E	Y	L	T	A	E	I	L	E	L	A	G	N	A	A	R	D	N	K	K	T	R	I	I	P	R	H	L	Q	L	A	I	R	N	D	E	E	L	N	K	L	L	G
353	Q96QV6	H2A1A_HUMAN	L	E	Y	L	T	A	E	I	L	E	L	A	G	N	A	S	R	D	N	K	K	T	R	I	I	P	R	H	L	Q	L	A	I	R	N	D	E	E	L	N	K	L	L	G
366	P04908	H2A1B_HUMAN	L	E	Y	L	T	A	E	I	L	E	L	A	G	N	A	A	R	D	N	K	K	T	R	I	I	P	R	H	L	Q	L	A	I	R	N	D	E	E	L	N	K	L	L	G
379	Q93077	H2A1C_HUMAN	L	E	Y	L	T	A	E	I	L	E	L	A	G	N	A	A	R	D	N	K	K	T	R	I	I	P	R	H	L	Q	L	A	I	R	N	D	E	E	L	N	K	L	L	G

Info Page | Histone Modification List | Human Histone List | #H1 | #H2A | #H2B | #H3 | #H4 | Entries Featured in Article

N: iPTMnet https://research.bioinformatics.udel.edu/iptmnet/

P: PhosphoSitePlus https://www.phosphosite.org/

H: HISTome2 http://www.actrec.gov.in/histome2

U: UniProtKB (Swiss-Prot) https://www.uniprot.org/

SCENERY B:

Investigation of the identified sites for 2-hydroxyisobutyrylation (hib) on H2A histone variants.

Step 1. Confirm the target modification is in the "Histone Modification List" (worksheet).

Step 2. Locate the target histone variants in the corresponding family "#H1/H2A/H2B/H3/H4" (worksheet).

Step 3. Click on the “2" button on the top left of the screen to access the information of all modification entries in the corresponding family.

Step 4. Search for the target modification at the beginning of each line of entries, and locate the entries for this modification on sites.

Step 5. Check the annotations of knowledgebases/databases or articles denoting the sources of information, search for the original page on the corresponding knowledgebases/databases or articles for more details.

Step 1. Confirm the target modification is in the "Histone Modification List" (sheet).

	A	B	C	D	E	F
1	**Annotation**	**Full Name**	**Amino Acid Residue with the Identified Modification**	**Modification Subfamily**	**Modification Family**	
2	*as follows*	acetylation	*as follows*	acetylation	acylation	
3	N-ac	*N*-terminal acetylation	*N*-terminus (M, S, T, P)	*as above*	*as above*	
4	ac	N^{ε}-acetylation	K	*as above*	*as above*	
5	but	butyrylation	K	butyrylation	acylation	
6	cro	crotonylation	K	crotonylation	acylation	
7	for	formylation	K	formylation	acylation	
8	glu	glutarylation	K	glutarylation	acylation	
9	bhb	*β*-hydroxybutyrylation	K	*β*-hydroxybutyrylation	acylation	
10	hib	2-hydroxyisobutyrylation	K	2-hydroxyisobutyrylation	acylation	
11	lac	lactylation	K	lactylation	acylation	
12	mal	malonylation	K	malonylation	acylation	
13	pal	*S*-palmitoylation	C	palmitoylation	acylation	
14	pro	propionylation	K	propionylation	acylation	
15	suc	succinylation	K	succinylation	acylation	
16	ADPr	ADP-ribosylation	K, R, S, E	ADP-ribosylation	ADP-ribosylation	
17	PAR	polyADP-ribosylation (PARylation)	K, R, S, E	ADP-ribosylation	ADP-ribosylation	
18	*as follows*	methylation	*as follows*	methylation	alkylation	
19	N-me3	*N*-terminal trimethylation	*N*-terminus (G)	*as above*	*as above*	
20	me (K)	monomethylation	K	*as above*	*as above*	
21	me2	dimethylation	K	*as above*	*as above*	
22	me3	trimethylation	K	*as above*	*as above*	

Info Page | Histone Modification List | Human Histone List | #H1 | #H2A | #H2B | #H3 | #H4 | Entries Featured in Article

Step 2. Locate the target histone variants in the corresponding family "#H1/H2A/H2B/H3/H4" (worksheet).

	A	B	C	D	E	F	G	H	I	J	K	L	M	N	O	P	Q	R	S	T	U	V	W	X	Y	Z	AA	AB	AC	AD	AE	AF	AG	AH	AI	AJ	AK	AL	AM	AN	AO	AP	AQ	AR	AS	AT
31																																														
32	**UniProt Accession**																																													
33	P0C0S8	H2A1_HUMAN	-	-	-	-	M	S	G	R	G	K	Q	G	G	K	A	R	A	K	A	K	T	R	S	S	R	A	G	L	Q	F	P	V	G	R	V	H	R	L	L	R	K	G	-	N
50	Q96QV6	H2A1A_HUMAN	-	-	-	-	M	S	G	R	G	K	Q	G	G	K	A	R	A	K	S	K	S	R	S	S	R	A	G	L	Q	F	P	V	G	R	I	H	R	L	L	R	K	G	-	N
67	P04908	H2A1B_HUMAN	-	-	-	-	M	S	G	R	G	K	Q	G	G	K	A	R	A	K	A	K	T	R	S	S	R	A	G	L	Q	F	P	V	G	R	V	H	R	L	L	R	K	G	-	N
84	Q93077	H2A1C_HUMAN	-	-	-	-	M	S	G	R	G	K	Q	G	G	K	A	R	A	K	A	K	S	R	S	S	R	A	G	L	Q	F	P	V	G	R	V	H	R	L	L	R	K	G	-	N
101	P20671	H2A1D_HUMAN	-	-	-	-	M	S	G	R	G	K	Q	G	G	K	A	R	A	K	A	K	T	R	S	S	R	A	G	L	Q	F	P	V	G	R	V	H	R	L	L	R	K	G	-	N
118	Q96KK5	H2A1H_HUMAN	-	-	-	-	M	S	G	R	G	K	Q	G	G	K	A	R	A	K	A	K	T	R	S	S	R	A	G	L	Q	F	P	V	G	R	V	H	R	L	L	R	K	G	-	N
135	Q99878	H2A1J_HUMAN	-	-	-	-	M	S	G	R	G	K	Q	G	G	K	A	R	A	K	A	K	T	R	S	S	R	A	G	L	Q	F	P	V	G	R	V	H	R	L	L	R	K	G	-	N
152	Q6FI13	H2A2A_HUMAN	-	-	-	-	M	S	G	R	G	K	Q	G	G	K	A	R	A	K	A	K	S	R	S	S	R	A	G	L	Q	F	P	V	G	R	V	H	R	L	L	R	K	G	-	N
170	Q8IUE6	H2A2B_HUMAN	-	-	-	-	M	S	G	R	G	K	Q	G	G	K	A	R	A	K	A	K	S	R	S	S	R	A	G	L	Q	F	P	V	G	R	V	H	R	L	L	R	K	G	-	N
187	Q16777	H2A2C_HUMAN	-	-	-	-	M	S	G	R	G	K	Q	G	G	K	A	R	A	K	A	K	S	R	S	S	R	A	G	L	Q	F	P	V	G	R	V	H	R	L	L	R	K	G	-	N
205	Q7L7L0	H2A3_HUMAN	-	-	-	-	M	S	G	R	G	K	Q	G	G	K	A	R	A	K	A	K	S	R	S	S	R	A	G	L	Q	F	P	V	G	R	V	H	R	L	L	R	K	G	-	N
222	Q9BTM1	H2AJ_HUMAN	-	-	-	-	M	S	G	R	G	K	Q	G	G	K	V	R	A	K	A	K	S	R	S	S	R	A	G	L	Q	F	P	V	G	R	V	H	R	L	L	R	K	G	-	N
239	P16104	H2AX_HUMAN	-	-	-	-	M	S	G	R	G	K	T	G	G	K	A	R	A	K	A	K	S	R	S	S	R	A	G	L	Q	F	P	V	G	R	V	H	R	L	L	R	K	G	-	H
256	O75367-2	H2AY_HUMAN	-	-	-	-	M	S	S	R	-	-	-	G	G	K	K	K	S	T	K	T	S	R	S	A	K	A	G	V	I	F	P	V	G	R	M	L	R	Y	I	K	K	G	-	H
275	Q9P0M6	H2AW_HUMAN	-	-	-	-	M	S	G	R	-	-	-	S	G	K	K	K	M	S	K	L	S	R	S	A	R	A	G	V	I	F	P	V	G	R	L	M	R	Y	L	K	K	G	-	T
292	P0C5Y9	H2AB1_HUMAN	M	P	R	R	R	R	R	R	G	S	S	G	A	G	G	R	G	R	T	C	S	R	T	V	R	A	E	L	S	F	S	V	S	Q	V	E	R	S	L	R	E	G	-	H
296	P0C5Z0	H2AB2_HUMAN	M	P	R	R	R	R	R	R	G	S	S	G	A	G	G	R	G	R	T	C	S	R	T	V	R	A	E	L	S	F	S	V	S	Q	V	E	R	S	L	R	E	G	-	H
300	A0A3B3IU63	H2AL3_HUMAN	-	-	M	A	G	N	K	M	F	C	R	-	-	P	R	R	Q	R	L	-	S	H	S	R	R	A	E	L	Q	F	P	V	S	H	L	E	R	C	L	R	E	S	Q	H
307	Q71UI9	H2AV_HUMAN	-	-	M	A	G	G	K	A	G	K	D	S	G	K	A	K	A	K	A	V	S	R	S	Q	R	A	G	L	Q	F	P	V	G	R	I	H	R	H	L	K	T	R	T	T
319	P0C0S5	H2AZ_HUMAN	-	-	M	A	G	G	K	A	G	K	D	S	G	K	A	K	T	K	A	V	S	R	S	Q	R	A	G	L	Q	F	P	V	G	R	I	H	R	H	L	K	S	R	T	T
332	O75409	HYPM_HUMAN	-	-	M	S	E	K	K	N	C	K	-	N	S	S	T	N	N	N	Q	T	Q	D	P	S	R	N	E	L	Q	V	P	R	S	F	V	D	R	V	V	Q	D	E	R	D
336																																														
337	**UniProt Accession**																																													
338	P0C0S8	H2A1_HUMAN	L	E	Y	L	T	A	E	I	L	E	L	A	G	N	A	A	R	D	N	K	K	T	R	I	I	P	R	H	L	Q	L	A	I	R	N	D	E	E	L	N	K	L	L	G
353	Q96QV6	H2A1A_HUMAN	L	E	Y	L	T	A	E	I	L	E	L	A	G	N	A	S	R	D	N	K	K	T	R	I	I	P	R	H	L	Q	L	A	I	R	N	D	E	E	L	N	K	L	L	G
366	P04908	H2A1B_HUMAN	L	E	Y	L	T	A	E	I	L	E	L	A	G	N	A	A	R	D	N	K	K	T	R	I	I	P	R	H	L	Q	L	A	I	R	N	D	E	E	L	N	K	L	L	G
379	Q93077	H2A1C_HUMAN	L	E	Y	L	T	A	E	I	L	E	L	A	G	N	A	A	R	D	N	K	K	T	R	I	I	P	R	H	L	Q	L	A	I	R	N	D	E	E	L	N	K	L	L	G
392	P20671	H2A1D_HUMAN	L	E	Y	L	T	A	E	I	L	E	L	A	G	N	A	A	R	D	N	K	K	T	R	I	I	P	R	H	L	Q	L	A	I	R	N	D	E	E	L	N	K	L	L	G
407	Q96KK5	H2A1H_HUMAN	L	E	Y	L	T	A	E	I	L	E	L	A	G	N	A	A	R	D	N	K	K	T	R	I	I	P	R	H	L	Q	L	A	I	R	N	D	E	E	L	N	K	L	L	G
422	Q99878	H2A1J_HUMAN	L	E	Y	L	T	A	E	I	L	E	L	A	G	N	A	A	R	D	N	K	K	T	R	I	I	P	R	H	L	Q	L	A	I	R	N	D	E	E	L	N	K	L	L	G
437	Q6FI13	H2A2A_HUMAN	L	E	Y	L	T	A	E	I	L	E	L	A	G	N	A	A	R	D	N	K	K	T	R	I	I	P	R	H	L	Q	L	A	I	R	N	D	E	E	L	N	K	L	L	G
452	Q8IUE6	H2A2B_HUMAN	L	E	Y	L	T	A	E	I	L	E	L	A	G	N	A	A	R	D	N	K	K	T	R	I	I	P	R	H	L	Q	L	A	V	R	N	D	E	E	L	N	K	L	L	G
465	Q16777	H2A2C_HUMAN	L	E	Y	L	T	A	E	I	L	E	L	A	G	N	A	A	R	D	N	K	K	T	R	I	I	P	R	H	L	Q	L	A	I	R	N	D	E	E	L	N	K	L	L	G
480	Q7L7L0	H2A3_HUMAN	L	E	Y	L	T	A	E	I	L	E	L	A	G	N	A	A	R	D	N	K	K	T	R	I	I	P	R	H	L	Q	L	A	I	R	N	D	E	E	L	N	K	L	L	G
493	Q9BTM1	H2AJ_HUMAN	L	E	Y	L	T	A	E	I	L	E	L	A	G	N	A	A	R	D	N	K	K	T	R	I	I	P	R	H	L	Q	L	A	I	R	N	D	E	E	L	N	K	L	L	G
508	P16104	H2AX_HUMAN	L	E	Y	L	T	A	E	I	L	E	L	A	G	N	A	A	R	D	N	K	K	T	R	I	I	P	R	H	L	Q	L	A	I	R	N	D	E	E	L	N	K	L	L	G
521	O75367-2	H2AY_HUMAN	L	E	Y	L	T	A	E	I	L	E	L	A	G	N	A	A	R	D	N	K	K	G	R	V	T	P	R	H	I	L	L	A	V	A	N	D	E	E	L	N	Q	L	L	K
528	Q9P0M6	H2AW_HUMAN	I	E	Y	L	A	A	E	I	L	E	L	A	G	N	A	A	R	D	N	K	K	A	R	I	A	P	R	H	I	L	L	A	V	A	N	D	E	E	L	N	Q	L	L	K
535	P0C5Y9	H2AB1_HUMAN	I	E	Y	L	T	A	K	V	P	E	L	A	G	N	E	A	Q	N	S	G	E	R	N	I	T	P	L	L	L	D	M	V	V	H	N	D	R	L	L	S	T	L	F	N
539	P0C5Z0	H2AB2_HUMAN	I	E	Y	L	T	A	K	V	L	E	L	A	G	N	E	A	Q	N	S	G	E	R	N	I	T	P	L	L	L	D	M	V	V	H	N	D	R	L	L	S	T	L	F	N
543	A0A3B3IU63	H2AL3_HUMAN	L	E	Y	L	T	A	N	I	[illegible]	E	K	V	G	K	E	V	K	N	S	C	R	L	C	I	T	P	E	H	V	K	R	A	L	Q	K	D	E	Q	L	R	W	I	L	E
547	Q71UI9	H2AV_HUMAN	L	E	Y	L	T	A	E	V	L	E	L	A	G	N	A	S	K	D	L	K	V	K	R	I	T	P	R	H	L	Q	L	A	I	R	G	D	E	E	L	D	S	L	I	-
559	P0C0S5	H2AZ_HUMAN	L	E	Y	L	T	A	E	V	[illegible]	E	L	A	G	N	A	S	K	D	L	K	V	K	R	I	T	P	R	H	L	Q	L	A	I	R	G	D	E	E	L	D	S	L	I	-

Info Page | Histone Modification List | Human Histone List | #H1 | #H2A | #H2B | #H3 | #H4 | Entries Featured in Article

Step 3. Click on the “2" button on the top left of the screen to access the information of all modification entries in the corresponding family.

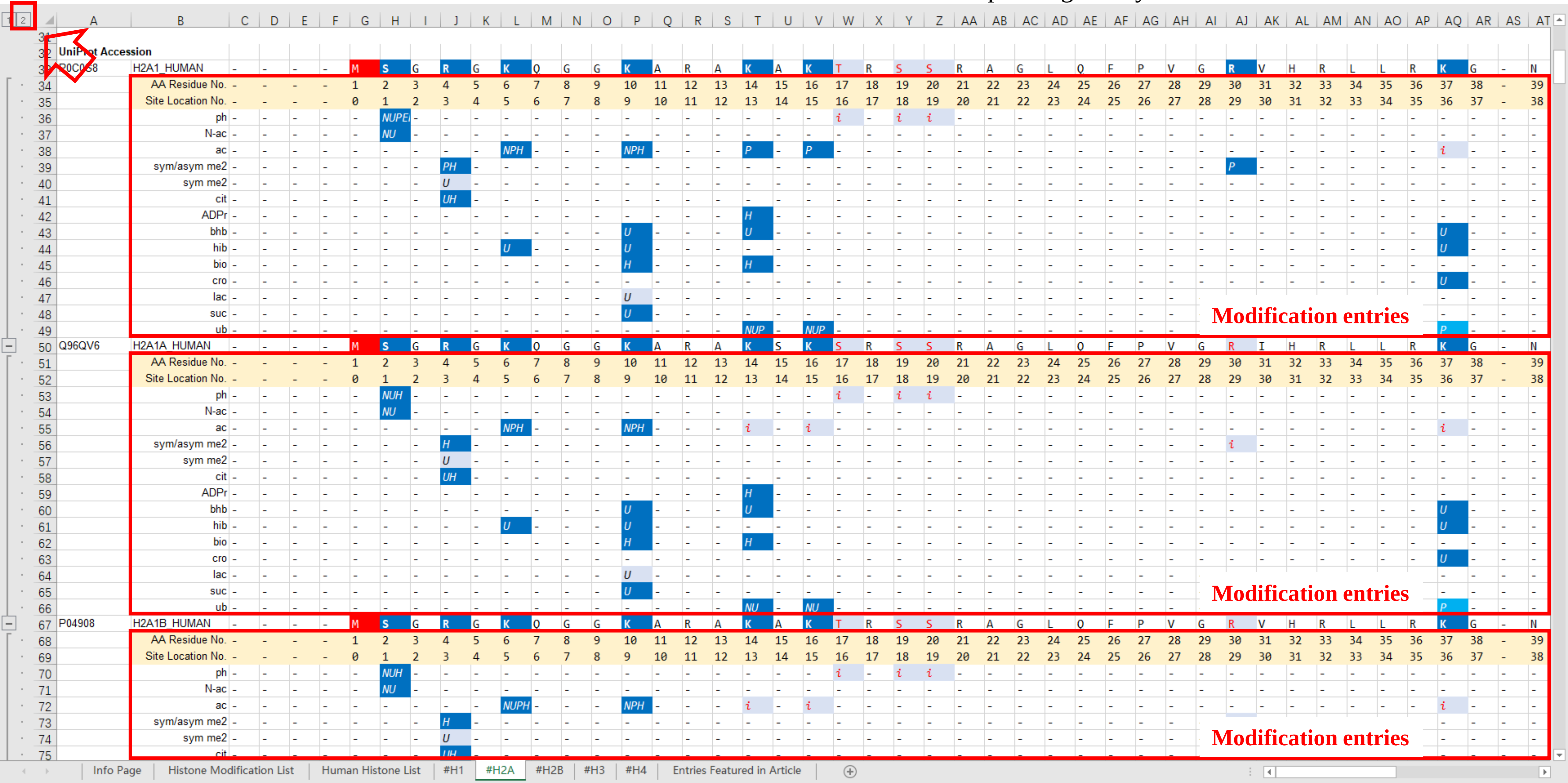

Step 4. Search for the target modification at the beginning of each line of entries, and locate the entries for this modification on sites.

	A	B	C	D	E	F	G	H	I	J	K	L	M	N	O	P	Q	R	S	T	U	V	W	X	Y	Z	AA	AB	AC	AD	AE	AF	AG	AH	AI	AJ	AK	AL	AM	AN	AO	AP	AQ	AR	AS	AT
31																																														
32	**UniProt Accession**																																													
33	P0C0S8	H2A1_HUMAN	-	-	-	-	M	S	G	R	G	K	Q	G	G	K	A	R	A	K	A	K	T	R	S	S	R	A	G	L	Q	F	P	V	G	R	V	H	R	L	L	R	K	G	-	N
34		AA Residue No.	-	-	-	-	1	2	3	4	5	6	7	8	9	10	11	12	13	14	15	16	17	18	19	20	21	22	23	24	25	26	27	28	29	30	31	32	33	34	35	36	37	38	-	39
35		Site Location No.	-	-	-	-	0	1	2	3	4	5	6	7	8	9	10	11	12	13	14	15	16	17	18	19	20	21	22	23	24	25	26	27	28	29	30	31	32	33	34	35	36	37	-	38
36		ph	-	-	-	-	-	NUPE	-	-	-	-	-	-	-	-	-	-	-	-	-	-	i	-	i	i	-	-	-	-	-	-	-	-	-	-	-	-	-	-	-	-	-	-	-	-
37		N-ac	-	-	-	-	-	NU	-	-	-	-	-	-	-	-	-	-	-	-	-	-	-	-	-	-	-	-	-	-	-	-	-	-	-	-	-	-	-	-	-	-	-	-	-	-
38		ac	-	-	-	-	-	-	-	-	-	NPH	-	-	-	NPH	-	-	-	P	-	P	-	-	-	-	-	-	-	-	-	-	-	-	-	-	-	-	-	-	-	-	i	-	-	-
39		sym/asym me2	-	-	-	-	-	-	-	PH	-	-	-	-	-	-	-	-	-	-	-	-	-	-	-	-	-	-	-	-	-	-	-	-	-	P	-	-	-	-	-	-	-	-	-	-
40		sym me2	-	-	-	-	-	-	-	U	-	-	-	-	-	-	-	-	-	-	-	-	-	-	-	-	-	-	-	-	-	-	-	-	-	-	-	-	-	-	-	-	-	-	-	-
41		cit	-	-	-	-	-	-	-	UH	-	-	-	-	-	-	-	-	-	-	-	-	-	-	-	-	-	-	-	-	-	-	-	-	-	-	-	-	-	-	-	-	-	-	-	-
42		ADPr	-	-	-	-	-	-	-	-	-	-	-	-	-	-	-	-	-	H	-	-	-	-	-	-	-	-	-	-	-	-	-	-	-	-	-	-	-	-	-	-	-	-	-	-
43		bhb	-	-	-	-	-	-	-	-	-	-	-	-	-	U	-	-	-	U	-	-	-	-	-	-	-	-	-	-	-	-	-	-	-	-	-	-	-	-	-	-	U	-	-	-
44		hib	-	-	-	-	-	-	-	-	-	U	-	-	-	U	-	-	-	-	-	-	-	-	-	-	-	-	-	-	-	-	-	-	-	-	-	-	-	-	-	-	U	-	-	-
45		bio	-	-	-	-	-	-	-	-	-	-	-	-	-	H	-	-	-	H	-	-	-	-	-	-	-	-	-	-	-	-	-	-	-	-	-	-	-	-	-	-	-	-	-	-
46		cro	-	-	-	-	-	-	-	-	-	-	-	-	-	-	-	-	-	-	-	-	-	-	-	-	-	-	-	-	-	-	-	-	-	-	-	-	-	-	-	-	U	-	-	-
47		lac	-	-	-	-	-	-	-	-	-	-	-	-	-	U	-	-	-	-	-	-	-	-	-	[illegible]	[illegible]	[illegible]	[illegible]	[illegible]	[illegible]	[illegible]	[illegible]	[illegible]	[illegible]	[illegible]	[illegible]	[illegible]	[illegible]	-	-	-	-	-	-	-
48		suc	-	-	-	-	-	-	-	-	-	-	-	-	-	U	-	-	-	-	-	-	-	-	[illegible]	[illegible]	[illegible]	[illegible]	[illegible]	[illegible]	[illegible]	[illegible]	[illegible]	[illegible]	[illegible]	[illegible]	[illegible]	[illegible]	[illegible]	-	-	-	-	-	-	-
49		ub	-	-	-	-	-	-	-	-	-	-	-	-	-	-	-	-	-	NUP	-	NUP	-	-	-	-	-	-	-	-	-	-	-	-	-	-	-	-	-	-	-	-	P	-	-	-
50	Q96QV6	H2A1A_HUMAN	-	-	-	-	M	S	G	R	G	K	Q	G	G	K	A	R	A	K	S	K	S	R	S	S	R	A	G	L	Q	F	P	V	G	R	I	H	R	L	L	R	K	G	-	N
51		AA Residue No.	-	-	-	-	1	2	3	4	5	6	7	8	9	10	11	12	13	14	15	16	17	18	19	20	21	22	23	24	25	26	27	28	29	30	31	32	33	34	35	36	37	38	-	39
52		Site Location No.	-	-	-	-	0	1	2	3	4	5	6	7	8	9	10	11	12	13	14	15	16	17	18	19	20	21	22	23	24	25	26	27	28	29	30	31	32	33	34	35	36	37	-	38
53		ph	-	-	-	-	-	NUH	-	-	-	-	-	-	-	-	-	-	-	-	-	-	i	-	i	i	-	-	-	-	-	-	-	-	-	-	-	-	-	-	-	-	-	-	-	-
54		N-ac	-	-	-	-	-	NU	-	-	-	-	-	-	-	-	-	-	-	-	-	-	-	-	-	-	-	-	-	-	-	-	-	-	-	-	-	-	-	-	-	-	-	-	-	-
55		ac	-	-	-	-	-	-	-	-	-	NPH	-	-	-	NPH	-	-	-	i	-	i	-	-	-	-	-	-	-	-	-	-	-	-	-	-	-	-	-	-	-	-	i	-	-	-
56		sym/asym me2	-	-	-	-	-	-	-	H	-	-	-	-	-	-	-	-	-	-	-	-	-	-	-	-	-	-	-	-	-	-	-	-	-	i	-	-	-	-	-	-	-	-	-	-
57		sym me2	-	-	-	-	-	-	-	U	-	-	-	-	-	-	-	-	-	-	-	-	-	-	-	-	-	-	-	-	-	-	-	-	-	-	-	-	-	-	-	-	-	-	-	-
58		cit	-	-	-	-	-	-	-	UH	-	-	-	-	-	-	-	-	-	-	-	-	-	-	-	-	-	-	-	-	-	-	-	-	-	-	-	-	-	-	-	-	-	-	-	-
59		ADPr	-	-	-	-	-	-	-	-	-	-	-	-	-	-	-	-	-	H	-	-	-	-	-	-	-	-	-	-	-	-	-	-	-	-	-	-	-	-	-	-	-	-	-	-
60		bhb	-	-	-	-	-	-	-	-	-	-	-	-	-	U	-	-	-	U	-	-	-	-	-	-	-	-	-	-	-	-	-	-	-	-	-	-	-	-	-	-	U	-	-	-
61		hib	-	-	-	-	-	-	-	-	-	U	-	-	-	U	-	-	-	-	-	-	-	-	-	-	-	-	-	-	-	-	-	-	-	-	-	-	-	-	-	-	U	-	-	-
62		bio	-	-	-	-	-	-	-	-	-	-	-	-	-	H	-	-	-	H	-	-	-	-	-	-	-	-	-	-	-	-	-	-	-	-	-	-	-	-	-	-	-	-	-	-
63		cro	-	-	-	-	-	-	-	-	-	-	-	-	-	-	-	-	-	-	-	-	-	-	-	-	-	-	-	-	-	-	-	-	-	-	-	-	-	-	-	-	U	-	-	-
64		lac	-	-	-	-	-	-	-	-	-	-	-	-	-	U	-	-	-	-	-	-	-	-	-	[illegible]	[illegible]	[illegible]	[illegible]	[illegible]	[illegible]	[illegible]	[illegible]	[illegible]	[illegible]	[illegible]	[illegible]	[illegible]	-	-	-	-	-	-	-	-
65		suc	-	-	-	-	-	-	-	-	-	-	-	-	-	U	-	-	-	-	-	-	-	-	-	[illegible]	[illegible]	[illegible]	[illegible]	[illegible]	[illegible]	[illegible]	[illegible]	[illegible]	[illegible]	[illegible]	[illegible]	[illegible]	-	-	-	-	-	-	-	-
66		ub	-	-	-	-	-	-	-	-	-	-	-	-	-	-	-	-	-	NU	-	NU	-	-	-	-	-	-	-	-	-	-	-	-	-	-	-	-	-	-	-	-	P	-	-	-
67	P04908	H2A1B_HUMAN	-	-	-	-	M	S	G	R	G	K	Q	G	G	K	A	R	A	K	A	K	T	R	S	S	R	A	G	L	Q	F	P	V	G	R	V	H	R	L	L	R	K	G	-	N
68		AA Residue No.	-	-	-	-	1	2	3	4	5	6	7	8	9	10	11	12	13	14	15	16	17	18	19	20	21	22	23	24	25	26	27	28	29	30	31	32	33	34	35	36	37	38	-	39
69		Site Location No.	-	-	-	-	0	1	2	3	4	5	6	7	8	9	10	11	12	13	14	15	16	17	18	19	20	21	22	23	24	25	26	27	28	29	30	31	32	33	34	35	36	37	-	38
70		ph	-	-	-	-	-	NUH	-	-	-	-	-	-	-	-	-	-	-	-	-	-	i	-	i	i	-	-	-	-	-	-	-	-	-	-	-	-	-	-	-	-	-	-	-	-
71		N-ac	-	-	-	-	-	NU	-	-	-	-	-	-	-	-	-	-	-	-	-	-	-	-	-	-	-	-	-	-	-	-	-	-	-	-	-	-	-	-	-	-	-	-	-	-
72		ac	-	-	-	-	-	-	-	-	-	NUPH	-	-	-	NPH	-	-	-	i	-	i	-	-	-	-	-	-	-	-	-	-	-	-	-	-	-	-	-	-	-	-	i	-	-	-
73		sym/asym me2	-	-	-	-	-	-	-	H	-	-	-	-	-	-	-	-	-	-	-	-	-	-	-	[illegible]	[illegible]	[illegible]	[illegible]	[illegible]	[illegible]	[illegible]	[illegible]	[illegible]	[illegible]	[illegible]	[illegible]	[illegible]	-	-	-	-	-	-	-	-
74		sym me2	-	-	-	-	-	-	-	U	-	-	-	-	-	-	-	-	-	-	-	-	-	-	-	[illegible]	[illegible]	[illegible]	[illegible]	[illegible]	[illegible]	[illegible]	[illegible]	[illegible]	[illegible]	[illegible]	[illegible]	[illegible]	-	-	-	-	-	-	-	-
75		cit	-	-	-	-	-	-	-	UH	-	-	-	-	-	-	-	-	-	-	-	-	-	-	-	-	-	-	-	-	-	-	-	-	-	-	-	-	-	-	-	-	-	-	-	-

hib sites on P0C0S8: K5, K9, K36, etc.

hib sites on Q96QV6: K5, K9, K36, etc.

… … … …

Info Page | Histone Modification List | Human Histone List | #H1 | #H2A | #H2B | #H3 | #H4 | Entries Featured in Article

Step 5. Check the annotations of knowledgebases/databases or articles denoting the sources of information, search for the original page on the corresponding knowledgebases/databases or articles for more details.

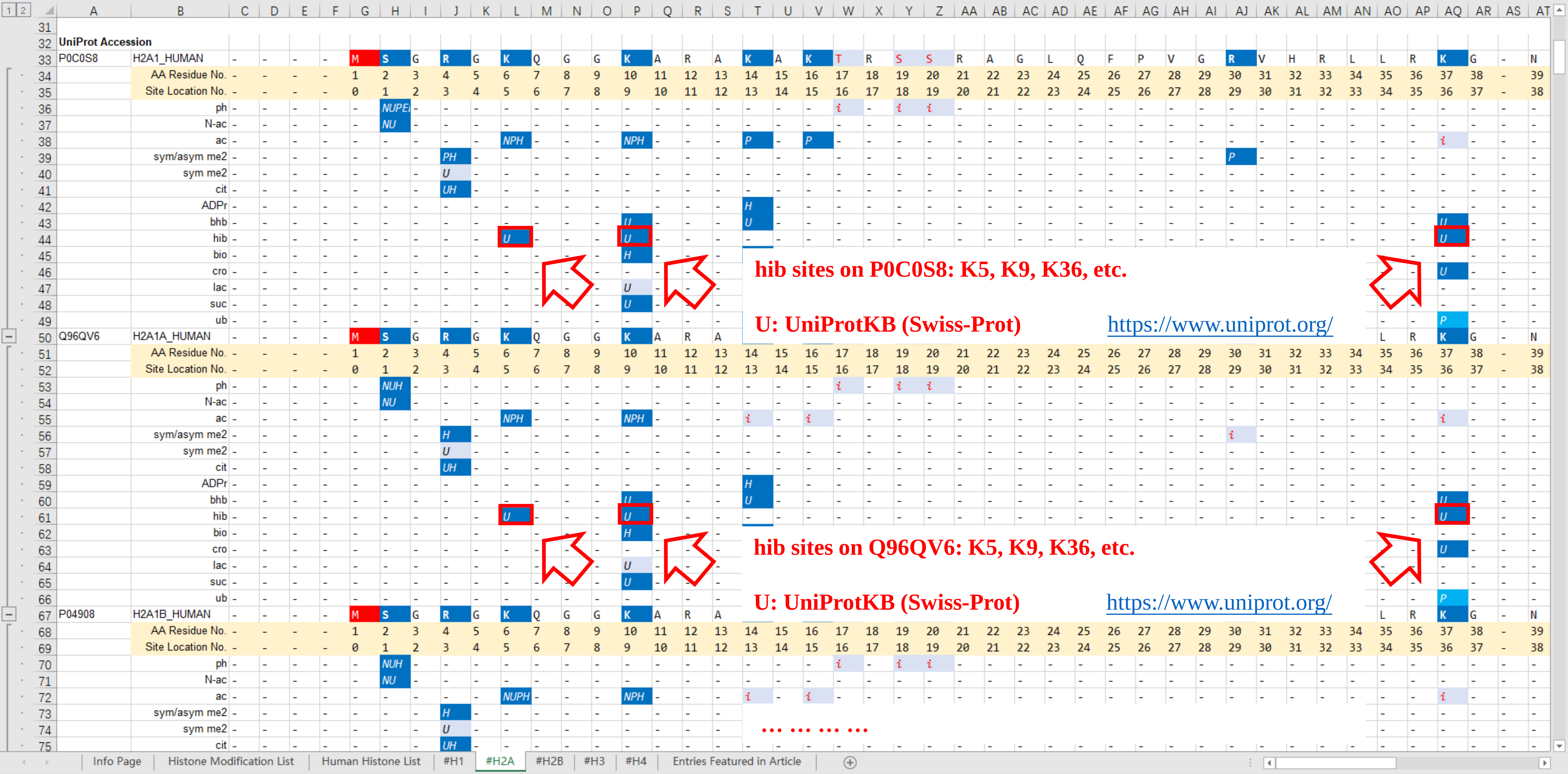

Supplement: Supplementary file 2 — User manual of Curated Catalogue of Human Histone Modifications (CHHM), (PDF). [file ijbsv20p3760s2.pdf]
